# Identification of Key Genes in the Response to * Salmonella enterica Enteritidis*, *Salmonella enterica Pullorum*, and Poly(I:C) in Chicken Spleen and Caecum

**DOI:** 10.1155/2014/154946

**Published:** 2014-02-23

**Authors:** Teng Ma, Guobin Chang, Rong Chen, Zhongwei Sheng, Aiqin Dai, Fei Zhai, Jianchao Li, Mingxiu Xia, Dengke Hua, Lu Xu, Hongzhi Wang, Jing Chen, Lu Liu, Guohong Chen

**Affiliations:** Animal Genetic Resources Laboratory, College of Animal Science and Technology, Yangzhou University, 88 South of University Avenue, Yangzhou, Jiangsu 225009, China

## Abstract

*Salmonella enterica Enteritidis* (*S. Enteritidis*) and *Salmonella enterica Pullorum* (*S. pullorum*) are regarded as a threat to poultry production. This study's aim is to characterize the expression profiles in response to three different challenges and to identify infection-related genes in the chicken spleen and caecum. Groups of the Chinese chicken breed Langshan were challenged with either *S. Enteritidis*, *S. pullorum*, or poly(I:C). The concentrations of cytokines and antibodies and the *Salmonella* colonization level of the caecum and liver were detected in each group at 7 days postinfection. Expression microarray experiments were conducted using mRNA isolated from both spleen and caecum. Crucial differentially expressed genes (DEGs) associated with immunity were identified. Four DEGs were identified in spleen of all three challenge groups (RBM16, FAH, SOX5, and RBM9) and different four genes in caecum (SOUL, FCN2, ANLN, and ACSL1). Expression profiles were clearly different among the three challenged groups. Genes enriched in the spleen of birds infected with *S. pullorum* were enriched in lymphocyte proliferation related pathways, but the enriched genes in the caecum of the same group were primarily enriched in innate immunity or antibacterial responses. The DEGs that appear across all three challenge groups might represent global response factors for different pathogens.

## 1. Introduction

*Salmonella* is frequently detected during the process of poultry production, especially *Salmonella enterica Enteritidis* (*S. Enteritidis*) and *Salmonella enterica Pullorum* (*S. pullorum*). Chicks over one-month of age have a low mortality rate when infected with *S. Enteritidis* [1], but in chicks less than one month old, this infection causes symptoms such as diarrhea, intestinal lesions, and an influx of heterophils into the gut accompanied by inflammation and damage to villi, leading to high mortality [2]. *S. pullorum* can cause young chicken cachexia, drop-head, wing prolapse, white fecal, loose stool, and anal blockage by excrement, with high mortality rates until two or three weeks of age [1].

*Salmonella* activates the innate immune system in the early stage of infection, particularly by LPS the TLR4 pathway after stimulation by lipopolysaccharide (LPS) [3]. Molecules involved in this process have been widely studied, including the TLR family and intermediate signal factors such as MyD88 [4]. However, compared with human or mouse, the studies of immune regulation mechanism on chicken were not sufficient. Most of these studies on chicken mainly focused on comprehensively investigating the expression patterns of a few crucial regulative genes after a specific infection [5].

To investigate the whole expression profiles of chicks infected with *Salmonella*, some researchers have performed expression microarray experiments and identified genes related to the innate immune response [6–11]. For instance, van Hemert et al. identified differential expression of genes by microarray experiments in intestinal tissue sampled from chicks infected with *S. Enteritidis* and uninfected individuals [8–10]. Similarly, Schokker et al. identified 20 key hub genes by analyzing a time series of microarray data of intestinal tissues collected at different time points after day-old chicks were infected with *S. Enteritidis* [11]. van Hemert et al. collected samples at the 1st, 3rd, 5th, 7th, 9th, 11th, 15th, and 21st day postinfection, and Schokker et al. sampled six consecutive time points postinfection, which were the 1st, 2nd, 4th, 8th, 12th, and 21st day postinfection. Considering the features of pathogenesis caused by *Salmonella*, we choose the 7th day postinfection as the sampling time point at which to carry out microarray experiments to investigate the immune response of the spleen and caecum to different types of *Salmonella* as well as Poly(I:C).

Poly(I:C) is regarded as an analog of RNA viruses such as avian influenza virus and is normally used as an immune-system activator, specifically activating the TLR3 pathway or the MDA5 pathway, which is related to antiviral function [12]. Its effect is different from LPS, which triggers the TLR4 pathway causing an inflammatory reaction. Therefore, the immune response to poly(I:C) might be extremely different from the response to these two serotypes of *Salmonella*, which could lead to a highly distinct gene expression profile [3, 8, 11, 13, 14].

We chose to study Langshan chickens in the current experiment. The Langshan breed has the good meat quality but a longer growth period than commercial broilers [15–17]. Te Pas et al. carried out a meta-analysis to integrate several individual artificial infection experiments and found that different chicken lines exhibited that different expression profiles of immune response in each line were varied [18], which was validated via Schokker et al.'s study as well [19]. Therefore, we hypothesized that because of different genetic backgrounds, the Langshan breed (Chinese indigenous) and commercial broiler breeds would have different expression profiles of antimicrobial response.

This study used three antigens to challenge day-old Langshan chicks. By comparing the gene expression profiles of the spleen and caecum of the challenged chicks at seventh days postinfection with the expression profiles of the control chicks at the same time, we identified DEGs that were involved in the immune response. These genes are candidates for breeding disease-resistant lines.

## 2. Materials and Methods

### 2.1. Preparation and Testing of Pathogenic Strains

*S. Pullorum* and *S. Enteritidis*, isolated from a local farm in Yangzhou, China, and then cultured for several generations, were used as pathogens to challenge day-old chicks. Bacteria were cultured in Luria-Bertani (LB) broth at 37°C for 12 h in a shaking incubator at 200 rpm/min before infection. The concentration of bacteria was diluted to 10^7^ cfu/mL.

Cloaca swabs were used to collect samples from two groups to test for the presence of *Salmonella* before the challenge. Each sample was cultured in 10 mL of LB broth at 37°C for 10 h and then plated on SS (*Salmonella Shigella*) agar at 37°C for 24 h before testing colonies with *Salmonella*-specific polyclonal antibodies (SSPA).

### 2.2. Challenging and Sample Collection

All experimental procedures were performed in accordance with the Guidelines for Experimental Animals established by the Ministry of Science and Technology (Beijing, China). Ninety Langshan chickens from the Rugao Langshan Chicken Genetic Resources Preservation Field were used. *S. Enteritidis* strains and *S. pullorum* strains were cultured in the liquid LB medium for 12 hours, and the bacterial concentration was determined via spectrophotometry before orally challenging the chicks. 10 mg/vial of poly(I:C) was purchased (Invivogen, San Diego, California, USA, CAS number: 31852-29-6 tlrl-pic). Chicks were fed with autoclave sterilizing chick feeds and divided into four groups, which were maintained in biological isolation. Twenty chicks were assigned to each of the three infected groups and 30 to the control group. All were maintained under uniform conditions of feeding and drinking ad libitum, at a temperature of approximately 33°C and 24-hour illumination. Twenty chicks were challenged with inoculum containing 10^7^ of *S. Enteritidis* via oral administration (“group SE”). Another 20 chicks were challenged with 10^7^ *S. pullorum* via oral administration (“group SP”). Another 20 chicks were challenged with 1 mL of poly(I:C) in diluent (1 mg/mL) via intramuscular injection into the thigh (“group poly(I:C)”). The control group of 30 chicks was orally perfused with 1 mL normal saline.

All surviving chicks were sacrificed at 7 days postinfection (DPI). Serum samples of those chicks were collected to determine the concentration of IgG, IgM, IL-1*α*, and IFN-*γ*. The spleen and caecum were isolated. After collecting the caecum content, they were placed into liquid nitrogen. Subsequently, spleen and caecum samples of four individuals from each group with intact experimental records were randomly selected and sent to Shanghai Biotechnology Co., Ltd. (SBC), which performed the microarray test.

### 2.3. Calculating *Salmonella* Load Counts in Caecum Content

After chicks were sacrificed, the caeca were completely scraped to collect the contents. After measuring the weight of the caecum contents, it was diluted in 100 mL normal saline. 1 mL diluent was further diluted 10-, 10^2^-, 10^3^-, 10^4^-, 10^5^-, and 10^6^-fold in 10 mL normal saline. Subsequently, 1 mL of the diluent of each concentration was directly plated on three plates of SS (*Salmonella Shigella*) agar at 37°C for 24 h. The appropriate concentration between 30 and 300 cfu/plate was generated from the 10^4^ dilution. The spread plate count method was used to count the number of *S. Pullorum* or *S. Enteritidis* colonies. The intestinal load capacity of *S. Pullorum* or *S. Enteritidis* was calculated from the formula:

(1) Bacteria  load(cfu/g) =Total  number  of  colonies/mL×104×100 mLWeight  of  caecum  content(g).

### 2.4. Determination and Statistical Analysis of the IL-1*α*, IFN*γ*, IgG, and IgM Content in Serum

Chicks were first sacrificed by cervical dislocation, then blood was immediately collected from heart. Subsequently; the bodies were dissected for collection of tissues. The collected blood without anticoagulant was incubated at 37°C for 2 h. Then, the blood was kept at 4°C overnight. After the serum naturally separated from the whole blood, this mixture was centrifuged at 3000 rpm/min for 10 min at 4°C. The upper serum layer was transferred into a new, sterile 1.5 mL centrifuge tube and stored at −80°C, and the blood cell layer was discarded. The IL-1*α*, IFN*γ*, IgG, and IgM content in the serum was determined with an enzyme-linked immunosorbent assay (ELISA) kit (Shanghai Yueyan Biotech Co., Ltd. Shanghai, China) according to the manufacturer's instructions. The absorbance in each well was determined via a microplate reader (NanoQant Infinite M200 PRO, TECAN, Männedorf, Switzerland). These raw results were used to fit a binomial regression line as a standard curve to calculate the IL-1*α*, IFN*γ*, IgG, and IgM content in serum samples. A one-way ANOVA was used to analyze all of the concentration data for each immunity index. The LSD algorithm was used for multiple comparisons. All analyses were carried out in Microsoft Excel 2010 (Microsoft Corporation (China), Beijing, China).

### 2.5. Identification of *Salmonella* in Spleen via Immunofluorescence

To investigate *Salmonella* invasion of tissues, spleens were collected for making frozen sections and stained with a fluorochrome-labeled polyclonal antibody. The chicken spleens were cut into 6 *μ*m thick slices by a freezing microtome (CM1950, Leica, Wetzlar, Germany), which were fixed with −20°C acetone for 10 min followed by washing with phosphate buffered saline (PBS) for 5 min three times and subsequently were incubated with 10% goat serum to block the unspecific antigens for 1 h then loading the diluent of *Salmonella*-specific polyclonal antibodies and incubating overnight. At the second day, after washing with PBS 5 min × 3 times, the secondary goat anti-mouse immunoglobulin (BOSTER, Wuhan, China) tagged with Cy3 fluorophore was added, incubated at 37°C for 1 h, and then washed with PBS 5 min × 3 times again. Subsequently, sections were incubated with 4′,6-diamidino-2-phenylindole (DAPI) (BOSTER, Wuhan, China) for 15 min at room temperature. Finally, the fluorescence anti-quenching agent (BOSTER, Wuhan, China) was added to sections, which were observed under an Inverted Fluorescence Microscopy system (IX71, OLYMPUS, Tokyo, Japan).

### 2.6. Microarray Hybridization Experiment

Four spleen samples and four caecum samples (from the same 4 individuals within each group) were sent to Shanghai Biotechnology Co., Ltd. (SBC, Shanghai, China) for the microarray hybridization. After strict total RNA extraction and quality control for each tissue sample following the standard protocol of SBC, two RNA samples from the same group were mixed and hybridized with one Agilent Chicken Whole Genome Expression chip (Agilent. SingleColor.26441). As a result, in each group there were two biological replications. All raw microarray data were normalized with the Quantile algorithm [20] via Gene Spring Software 11.5.1 (Agilent Technologies, Santa Clara, CA, US).

### 2.7. Filtering and Analyzing DEGs

Gene Spring Software 11.5.1 was adopted for further filtering of DEGs from normalized data through a series of quality control steps. At first, all detected probes were kept. The QC probes were removed. Two chips, one for male and the other for female, were used for each group as biological duplications. The following filtered data were created from the Advanced Analysis operation: significance analysis, corrected *P* value cut-off: 0.05; post hoc test: Tukey HSD; selected test: one-way ANOVA; *P* value computation: asymptotic; multiple testing correction: Benjamini-Hochberg. The expression data from the control group were used as the control. Primary DEGs with fold changes ≥1.5 and ≤0.75 were retained. Then those with fluorescence intensity more than 100 were kept as candidate genes from the results of clustering analysis carried out for all DEGs, and comparative analysis was performed with Gene Spring Software 11.5.1. Subsequently, DAVID (http://david.abcc.ncifcrf.gov/) [21] was used in the enrichment analysis of gene ontology (GO) and KEGG pathways.

### 2.8. qRT-PCR Validation of Microarray Data

Five genes with significant differential expression folds were selected to validate the reliability of the microarray data: RSFR, VPREB3, CD72, IL15, and GAL1 (see Table S7, in Supplementary Material available online at http://dx.doi.org/10.1155/2014/154946). The experimental design for the qRT-PCR was the same as that used for the chips, but each sample had four technical replicates. The same RNA samples that were used in the microarray experiment were used for qRT-PCR. The relative quantification of the RT-PCR experiment was performed according to the instructions of the PrimeScript RT Master Mix kit and SYBR Premix Ex Taq II kit (PrimeScript RT Master Mix (Perfect Real Time, DRR036); SYBR Premix Ex Taq II (Tli RNase H Plus, DRR820). The Applied Biosystems 7500 Real-Time PCR System was used for this experiment. REST was used to analyze the experimental results [22, 23].

## 3. Results and Analysis

### 3.1. Infection Status

By 7 days postinfection (7 DPI), when all chicks were sacrificed and sampled, the mortality of group SE was 15%, and the mortality of group SP was 10%. No chicks died in group poly(I:C) before they were sacrificed, which suggests that the dose of bacteria used to challenge day-old chicks was appropriate to stimulate immunoreaction but did not cause high mortality. Necropsy was carried out, and no obvious pathological changes were found. We inferred that these results were probably caused by bacteremia, which led to multiple organ failure. This study aimed to investigate the immunoreaction to *Salmonella* in vivo. Thus, dead individuals were removed and only live chicks were used for follow-up experiments. Chicks whose serum was not collected were also removed from this study.

### 3.2. Results of Determining *Salmonella* Counts in Caecum Content

The results (Table 1) indicate that *Salmonella* was identified in groups SE and SP. The microbial load of *Salmonella* in groups SE and SP was nearly 10^7^ cfu/g; there was no significant difference between the two groups. No *Salmonella* was found in the control and poly(I:C) group. *Salmonella* remained in the caecum until 7 DPI, which mean that immunoreaction might continue to occur.

### 3.3. IL-1*α*, IFN*γ*, IgG, and IgM Content in Serum

The level of two cytokines (IL-1*α*, IFN*γ*) and two antibodies (IgG, IgM) was determined via ELISA (Table 2). The mean IL-1*α* concentration of the control group was significantly lower than the poly(I:C) group. The mean IFN*γ* concentration of the control group was significantly lower than the SE group. Thus, a significant, though moderate, immune response could be detected at 7 DPI.

In terms of the adaptive immune response, the IgG level of group SP was significantly higher than that of the other groups. Although the average IgM level of the SP group was not significantly higher than the other groups, it was at least 10 mg/L more than other groups. The results show that chicks had different Ig response patterns to *S. Enteritidis* and the adaptive immune system of chicks challenged with *S. pullorum* was activated. To investigate the expression changes of this phenomenon, the microarray experiment was performed to identify differences at the mRNA level.

### 3.4. *Salmonella* Still Exists in Spleen at 7 DPI

**Figure 1:**
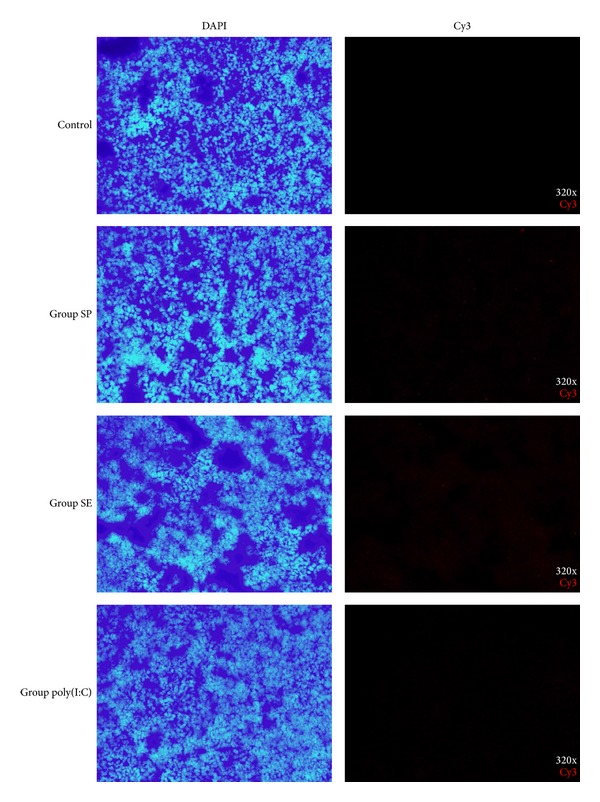
Typical immunofluorescence pictures of frozen sections. DAPI was used to stain the cellular nucleus, and polyclonal antibodies labeled by Cy3 were used to stain the bacteria body of *Salmonella*.

We also carried out immunofluorescence experiments on frozen sections of the chicken spleens. At 7 DPI *Salmonella* still could be found in the spleens of groups SP and SE (Figure 1). The number of bacteria in the SP group (total 3882.92 positive spots/mm^3^) was significantly greater than that of the SE group (total 1244.52 positive spots/mm^3^). The control group and the poly(I:C) group were free from *Salmonella*. This result suggests that *S. Pullorum* has a stronger tissue invasion ability than *S. Enteritidis* for the strains used in this experiment.

### 3.5. Statistical Description of DEGs of Spleen and Caecum in the SE, SP, and Poly(I:C) Groups

DEGs in the spleen with a *P* value < 0.05 and a FC (Fold Change) ≥1.5 for each group are shown in Table S8. The proportion of upregulated genes in group SP was greater than that in the other two treatment groups, which suggests that the immune response might be still active at 7 DPI, in agreement with the immune index. In contrast, the proportion of downregulated genes in the poly(I:C) group was slightly higher than the other two groups.

The DEGs in the caecum for each group were identified, and the statistics are shown in Table S9. Because poly(I:C), which was not administered orally, initiated immune stimulation through the blood rather than the intestinal track, we hypothesized that there would be fewer DEGs in the caecum than those of the other two groups, which was confirmed by the results (Table S9). In addition, the identified DEGs were not primarily enriched in immune processes. The proportion and the absolute quantity of DEGs in group SP were 16.21% greater than those in group SE, which indicates that immune response in the SP group was still active and the pathogenesis mechanisms were distinct between SP and SE groups.

### 3.6. Filtering and Analyzing DEGs in the Spleen

Through a series of filtering steps, the DEGs with high expression and high differential expression were obtained in group SP. There were 16 upregulated probes with |FC | ≥2, Fluorescence Signal, and Raw_Data > 100 (Table S1-1), of which 7 probes had annotation information (Table 3(a)). Some of these genes are reported to be correlated with immune regulation. RANGAP1 is a GTPase activator of RAN, and SLCO1B3 is an important membrane transport protein. VAV2 is an oncogene family member that is expressed in multiple tissues, unlike VAV1, which is only expressed in hematopoietic cells. Boettcher et al. found that, at early stages of the immune response, P+GC triggered the amplification cascade of the Cav1-Vav2-RhoA signal pathway dependent on phosphotyrosine, leading to the rearrangement of the cytoskeleton and blocking host cell phagocytosis of bacteria [24]. However, the function of the rest of the probes was unknown.

Ten downregulated genes (Table S1-2) were obtained from group SP, six of which were annotated (Table 3(b)). Some of these have been observed to play an important role in immune function. Nitto et al. reported that RSFR (leukocyte ribonuclease A-2) not only acted as a nuclease but also had a antibacterial activity [25]. The mutation of MERTK (Proto-oncogene tyrosine-protein kinase MER) was associated with blocking phagocytosis by retinal pigment epithelial cells and with retinitis pigmentosa [26]. Linger et al. found that MerTK, Nyk, and Tyro12 mediated pathologic platelet aggregation, macrophage-mediated clearance of apoptotic cells, release of cytokines, and differentiation and proliferation [27]. RAB32 is an important factor in autophagy in fruit flies and also in lipid deposition [28, 29], and the mutation of specific sites was linked to susceptibility to lepriasis [30]. In addition, it also mediated protein trafficking to lysosome-like organelles. Mutation of TPRN was associated with recessive progressive autosomal deafness syndrome [31]. TMEM140 may be involved in maintaining the activity of stem cells [32].

After filtering the array results of other two groups, there were 67 DEGs in group SE and 66 DEGs in the poly(I:C) group (Tables S2-1, S2-2, S3-1, and S3-2). Except for VPREB3 (upregulated) and RSFR (downregulated), the other genes were mainly involved in the circulatory system and hemocyte development. RSFR is an antimicrobial protein that was downregulated in the SE and poly(I:C) groups, which might reflect the repression of innate immunity system. VPREB3 was the most highly differentially expressed gene in the poly(I:C) group, about 7.5 times more than the control. It was reported that this gene encoded a protein that is homologous to mouse VpreB3 (8HS20), which is specifically expressed in every stage of B lymphocyte differentiation, and correlated with inchoate *μ* heavy chain, which was produced in the process of the generation of the B lymphocyte receptor. Rosnet et al. found that covalent VpreB3 binding with free IgLC results in the maintenance of IgLC in the endoplasmic reticulum instead of secreting it, but this did not influence the production of IgM, thereby modulating the immune reaction [33].

### 3.7. Filtering and Analyzing DEGs in the Caecum

Expression profiles in the caecum of group SP were determined using the procedures described above. Fifty-two upregulated probes (34 were annotated) and 18 downregulated probes (10 were annotated) were identified (Tables 4(a) and 4(b), Tables S4-1 and S4-2). Colonization of the caecum content was observed when the challenged group was sampled, indicating that the expression profile of that tissue should reflect the immune response. Wong et al. showed that FHL2, which upregulated in the current study, could regulate the expression of IL6 via the p38 and NF-*κ*B pathways and was involved in the protection of muscle cells and inflammatory response after damage [34]. However, other DEGs in the caecum were mainly involved in muscle development according to their annotation. For example, PDLIM3 is highly expressed in developing human skeletal muscle [35].

Among the downregulated genes, IFIT5 (interferon-induced protein with tetratricopeptide repeats 5) is a protein involved in immune response. Vanderven et al. studied the immune response to duck influenza and found that IFIT5 was upregulated 1000 times as much as the control [36]. In addition, other researchers found that this gene was associated with antivirus and antibacterial activity [37, 38]. Downregulation of this gene at 7 DPI suggests that the immune process was being downregulated and innate immune activity repressed. CATHL3, an antimicrobial peptide, was also identified. CD72, which was identified as the gene downregulated the most, was downregulated 12.87-fold. Alcón et al. reported that CD72 was a repressive receptor on the surface of the NK cells, which regulates the production of cytokines [39]. Downregulation of this gene might facilitate production of more cytokines in NK cells. There is minimal information regarding the effect of the other downregulated genes on the immune system. Given the fact that the IgG level of group SP was significantly higher than the other two treatment groups, it may be inferred that the intestine was still infected, but the chief mode of immune protection had shifted from cellular immunity to adaptive immunity.

The upregulated genes of the SE and poly(I:C) groups were not enriched in immune function; however, some downregulated genes in the SE and poly(I:C) groups were linked to immune response (Tables S5-1, S5-2, S6-1, and S6-2). For instance, RSFR (fold change = −5.33) and B-G (fold change = −10.26) were identified as downregulated genes in group SE. The B-G segment was identified by Miller et al. as an important variable area of chicken MHC [40], which is involved with antigen presentation. The downregulation of this gene indicates that the antigen presentation was repressed, potentially leading to lower expression than in the control group, while tissue reconstruction activity had begun.

### 3.8. Common DEGs in All Three Experimental Groups

**Figure 2:**
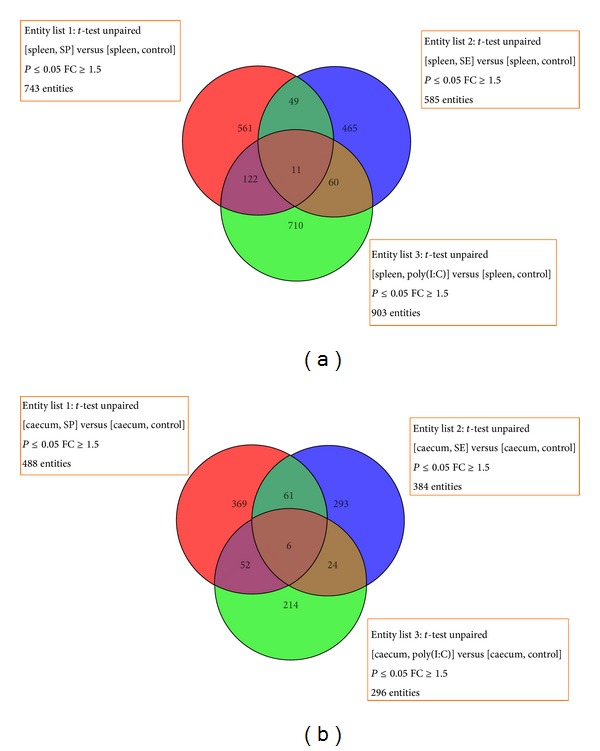
(a) Venn diagram of DEGs in the spleen of all three treatment groups. (b) Venn diagram of DEGs in the caecum of all three treatment groups.

A Venn diagram illustrates the differences and similarities among the three treatment groups (Figure 2). There were four annotated genes out of 11 DEGs present in all three groups in the spleen (Table 5). These four genes were: SOX5; an enhancement element of COL2A1, which modulated the activity of the promoter of the reverse transcription transposon known as LINE; RBM16 and RBM9, which are involved in mRNA editing; and FAH, which participates in the catabolism of tyrosine. Transcription was obviously active in the spleen, inferring that these four genes might play a role in immune response and development of the spleen.

There were four annotated genes of the six DEGs found in the caecum of the three treatment groups (Table 6). Two of these four genes, SOUL and ANLN, were upregulated. The SOUL protein is a new member of the BH3 protein family that triggers apoptosis via initiating the mitochondrial permeability transition [41]. The two downregulated genes were FCN2 and ACSL1. FCN2 indirectly activates the innate immune system via enhancing the activity of neutrophil granulocyte phagocytosis of *Salmonella Typhimurium* and is also regarded as an immune opsonin [42]. The downregulation of FCN2 indicated that innate immune activity might be repressed in the caecum 7 DPI.

Most DEGs were only found in one group, which demonstrated that, at 7 DPI, the three groups had very different expression profiles, reflecting the different physiological status of each treatment group.

### 3.9. GO and Pathway Enrichment Analysis of DEGs

GO and Pathway enrichment analyses of DEGs were carried out by using the online tool DAVID. DEGs in the SP group were clustered into 11 classes under medium parameters for Biology Process (BP) in GO (Table 7). The class with the highest enrichment value included immune response, development, and differentiation of lymphoid tissue and differentiation and activation of lymphoid cells, which was specific to the differentiation of T-cells and positive regulation of T-cell activity, but other classes were enriched in material and energy metabolism.

The same analyses were carried out on the SE and poly(I:C) groups. Clustering DEGs in group SE using the GO Biology Process showed enrichment in coagulation and wound-healing processes, which might be correlated with immune response leading to injury to the spleen. DEGs in the poly(I:C) group were primarily enriched in nucleic acid metabolism processes, which may indicate that poly(I:C) promotes the activity of nucleic acid metabolism-related pathways.

Furthermore, caecal DEGs of group SP were enriched in a cluster similar to the splenic DEGs of group SP (Table 8), mainly antibacterial processes and activation of antibacterial-related innate immune pathways for GO BP, membrane system and cytoskeletal components for GO CC, nucleic acid metabolism and ion-binding proteins for GO molecular function (MF).

As seen from Table 9, 11 DEGs were involved in the splenic immune response in group SP. According to the enrichment analysis of the caecum of the group SP, there were eight genes (Table 10) involved in the immune response to bacteria. However, not all of these genes had the same direction of expression. Some of them were upregulated in the spleen of group SP, whereas those DEGs in the caecum of group SP were primarily downregulated.

### 3.10. Results of qRT-PCR

Five genes selected from the above results (RSFR, IL15, VPREB3, CD72, and GAL1) were used for qRT-PCR to validate the reliability of microarray data. As depicted in Table 11, the results of qRT-PCR showed the same expression trend as the microarray data. VPREB3 was expressed in the spleen, and the transcription abundance in the challenged group at 7 DPI was significantly higher than the control group. This result suggests that VPREB3 expression is correlated with B cells migrating into the spleen, experiencing clonal selection and reaching maturity.

## 4. Discussion

*Salmonella* were present in the content of the caecum, and, therefore, bacterial colonization still existed in the intestine in challenged groups at 7 DPI. This experiment simulated the oral infection route of *Salmonella* in a chicken farming operation and suggests that a persistent infection could effectively stimulate the immune response.

According to the results of the serological Ig test, *S. pullorum* effectively activated adaptive immunity by the 7th day after the challenge, while the innate immune system had been repressed. Furthermore, the adaptive immunity index of the poly(I:C) group was the same as the control, whereas the innate immunity index of interleukin and interferon were higher than those of control. Typically, at the 7th day after *Salmonella* infection chicks mortalities would reach its peak, but in the present study, its infectious ability was not severe enough to lead to high mortality but did effectively activate the immune system [43]. Because the bacterial strains we chose were not the original field wild strains but strains cultured for several generations, their virulence had been weakened. The splenic expression profile of group SE did not reflect typical bacterial infection response and did not show relatively high level of IFN*γ*, which is involved in a complex regulation network including the response to highly pathogenic avian influenza viruses [44].

The spleen is a peripheral immune organ that plays a major role in immune response. The number of DEGs in the poly(I:C) group was greater than that of groups SE and SP. This suggested that poly(I:C) was an efficient immune activator when it was injected into the chick's thigh muscle, because it directly stimulated the spleen via transporting the poly(I:C) from the thigh muscle through the circulation.

It is of interest that the number of DEGs in the caecum was less than the number of DEGs found in the spleen. We inferred that, at 7 DPI, the normal function of the caecum was recovering from the active immune response. Because the poly(I:C) group did not directly stimulate the caecum, the number of DEGs was the lowest of all treatment groups. The 296 DEGs identified in the poly(I:C) group in the caecum were likely caused by differences in normal physiological status between individuals.

Both significantly upregulated genes and enriched clusters revealed the activity of the immune system in the spleen of group SP. At 7 DPI, group SP displayed effective activation of adaptive immunity given that the genes activating adaptive immunity were identified. Most upregulated genes were involved in the activation of B lymphocytes and the related adaptive immune function. The downregulated genes correlated with repression of the innate immune system and enhancement of the adaptive immune system in the SP group.

Based on the functions of those DEGs, identified in the spleen of the SP group, it may be inferred that, at 7 DPI, VAV2 was upregulated to reduce antigen uptake, and the transcription abundance of other genes involved in phagocytosis was downregulated. At early stages of response by the innate immune system, downregulation of these innate immunity-related genes suggests that they might have important functions and be highly expressed. Genes repressing innate immune activity may be crucial regulators of activating the adaptive immune system because the expression was evidently associated with both the innate and adaptive immune systems [45]. However, according to Boettcher's study, VpreB3 (8HS20) was not expressed in the spleen, which was contradictory to our result, which was validated by qRT-PCR. We hypothesize that this inconsistency may be due to differences in the mechanism of activation of the immune system in the chicken and mouse.

In the poly(I:C) group, the result of the serological test was not consistent with the characteristics of expression profile of the spleen and caecum. We hypothesize that because poly(I:C) was injected directly into the thigh muscle, it stimulated not only immune organs but also other tissues to produce an extended immune response and release more cytokines. This was an expected result, because poly(I:C) is known as a viral analog that mainly stimulates the antiviral pathway [14]. It was a different type of antigen from the two serotypes of *Salmonella*, which were live bacteria that produced a complex immune response, activating the innate immune system and subsequent adaptive immunity. Furthermore, the disease symptoms of *S. Enteritidis* and *S. pullorum* are distinct from each other [1], which means that some differences exist in the immune process, which were reflected in the microarray profiles.

The IgG level of group SP was higher than the other two treatment groups. Considering the results of the enrichment analysis of the expression profiles of each group, it was deduced that group SP remained in an immune reactive state, but group SE seemed to be recovering from the *S. Enteritidis* infection at 7 DPI. This distinction has been reported to be correlated with the different measures of pathogenic invasion and colonization [46]. *S. Enteritidis* mainly colonizes in the intestine, but *S. pullorum *can invade organs such as the liver and the spleen. This distinction makes eliminating *S. pullorum* from the internal tissue environment more difficult and makes *S. pullorum* tend to reinfect chickens more easily. The activity of nucleic acid metabolism due to a high level of cytidylate in the poly(I:C) group was easier to be observed in the microarray profile at 7 DPI.

According to enrichment results of group SP, innate immunity was still active at 7 DPI, especially T-cell maturity and differentiation processes or pathways. The GO MF of DEGs was enriched in membrane and cytoplasmic protein. Given that most immune cells possess many membrane signal receptors or adaptors, the enrichment in membrane proteins may suggest immune cell activity, which is also supported by the result from the enrichment analysis of the GO cell component (CC), which is chiefly enriched in metallopeptidases and ion-binding proteins relevant clusters. Interestingly, the results of enrichment clustering of MF and CC of DEGs of both the SE and the poly(I:C) groups were similar to that of the SP group. However, gene expression in the caecum of the SE and poly(I:C) groups was not enriched in the immune processes in spite of the similar enrichment results for CC and MF as compared to group SP.

The infection of *S. pullorum* was still active in the intestine and spleen, where active immune response could be identified via the microarray expression profiles. However, the other two groups seemed to have weaker immune responses given that the DEGs of those groups were enriched in tissue function-related pathways or processes. Therefore, the genes involved in the immune response of group SP according to the enrichment analysis became the focus of the study. Among those genes, several genes were reported to participate in an immune-related process or pathway. For instance, CD28 combines with CD80 to coactivate T-cells [47]; IL15 is an activation factor for lymphocytes and T-cells, activating the cytotoxic effect of NK cells [48]. However, both CD28 and IL15 were downregulated, which indicated that the differentiation of T-cells or mature of NK cells was repressed.

In our study, the expression profile in the spleen did not reflect the activation of T-cells and NK cells at 7 DPI. However, it has been observed by other researchers that upregulated IL7 promoted the proliferation of pro-B-cells [49]. Therefore, it was inferred that the reason that there was high IgG level in group SP but a low cytokine level (Table 2) was mainly because upregulated IL7 induced the maturation and proliferation of B lymphocytes to produce antibodies to protect against *S. pullorum*. Although other upregulated genes also promoted cell differentiation and proliferation, downregulated IRF8, which mediates apoptosis of cancerous nonhematopoietic cells via Fas, Bax, FLIP, Jak1, and STAT1, availed the proliferation of stem cells in the spleen [50, 51]. NF-kappaB is an important transcription factor in the innate immune system; its expression level is regulated by Notch1 through an intricate regulation pathway [52], and it is regulated by several upstream regulatory factors of MyD88 and regulates the transcription abundance of many cytokines [53]. The upregulation of Notch1 in our experiment suggested that the NF-kappaB pathway might be involved in cytokine release.

We also identified several immune-related DEGs from the enrichment results of the caecum in the group SP. However, only the 5-hydroxytryptamine (serotonin) receptor 2B, a neuropeptide, and adiponectin, a metabolism-regulating hormone [54], were upregulated. The other six genes were all downregulated. This situation indicated that the innate immune system was repressed, whereas adaptive and mucosal immunities might be enhanced in a challenged caecum at 7 DPI compared to normal caecal tissue. In addition, neither the 5-hydroxytryptamine (serotonin) receptor 2B nor adiponectin has been reported to be directly associated with immune response, but they might regulate immune function by an unknown mechanism via indirect signal transduction.

Some immune-related genes that were identified lacked functional information. Because of the differences between the immune systems of poultry and mammals, immune-related genes that exist in both might not be regulated in the same way [55–57]. The microarray analysis will be helpful for further study of the immune response of poultry to specific pathogens. At the same time, the identified genes could be used as candidates for breeding disease-resistant poultry.

Finally, we found that some highly expressed genes were not annotated, but some probes were aligned with some segments of bacterial genes with *E*-value ≤ 0.1 via the tBLASTx program, which hinted that these probes might correspond to some unknown immune response mechanism. Whether the sequences corresponding to those probes are correlated with coevolution of the immune system and pathogens or horizontal transmission of sequence segments of pathogens requires further research.

## 5. Conclusion

The expression profiles were distinctly different in each challenged group, indicating different pathologic processes. The DEGs of the group infected with *S. pullorum* in the spleen were enriched in lymphocyte proliferation-related pathways, which suggested that T cells or B cells were activated at 7 DPI. However, the DEGs in the same group in the caecum were enriched for innate immunity or antibacterial response, indicating that inflammation of the gut was still present. Some of these enriched genes are involved in promoting the immune response. Furthermore, the DEGs found across all three groups may represent global response factors to different pathogens. All identified DEGs warrant further investigation into their roles in the immune system and may potentially be utilized as candidate genes to assist in the breeding of disease-resistant lines, thereby reducing the need for use of antibiotics or medicines.

## Supplementary Material

Table S1-1: Upregulation of differentially expressed genes in the spleen in group SP with FC ≥ 2 and Signal Density of Raw Data ≥ 100.
Table S1-2: Downregulation of differentially expressed genes in the spleen in group SP with FC ≥ 2 and Signal Density of Raw Data ≥ 100.
Table S2-1: Upregulation of differentially expressed genes in the spleen in group SE with FC ≥ 2 and Signal Density of Raw Data ≥ 100.
Table S2-2: Downregulation of differentially expressed genes in the spleen in group SE with FC ≥ 2 and Signal Density of Raw Data ≥ 100.
Table S3-1: Upregulation of differentially expressed genes in the spleen in group Poly(I:C) with FC ≥ 2 and Signal Density of Raw Data ≥ 100.
Table S3-2: Downregulation of differentially expressed genes in the spleen in group Poly(I:C) with FC ≥ 2 and Signal Density of Raw Data ≥ 100.
Table S4-1: Upregulation of differentially expressed genes in the caecum in group SP with FC ≥ 2 and Signal Density of Raw Data ≥ 100.
Table S4-2: Downregulation of differentially expressed genes in the caecum in group SP with FC ≥ 2 and Signal Density of Raw Data ≥ 100.
Table S5-1: Upregulation of differentially expressed genes in the caecum in group SE with FC ≥ 2 and Signal Density of Raw Data ≥ 100.
Table S5-2: Downregulation of differentially expressed genes in the caecum in group SE with FC ≥ 2 and Signal Density of Raw Data ≥ 100.
Table S6-1: Upregulation of differentially expressed genes in the caecum in group Poly(I:C) with FC ≥ 2 and Signal Density of Raw Data ≥ 100.
Table S6-2: Downregulation of differentially expressed genes in the caecum in group Poly(I:C) with FC ≥ 2 and Signal Density of Raw Data ≥ 100.
Table S7: qRT-PCR primers for 5 target genes.
Table S8: Statistical description of the difference in the expression of genes in each group in the spleen.
Table S9: Statistical description of the difference in the expression of genes in each group in the caecum.

## Figures and Tables

**Table 1:** The *Salmonella* load in each group.

Groups	Microbial load of *Salmonella* in caecum (10^6^ cfu/g)
Group SE	163.75 ± 6.39
Group SP	171.45 ± 10.45
Poly(I:C)	0
Control	0

**Table 2:** Immune indices of the experimental groups.

Groups	IL1-*α* (ng/L)	IFN-*γ* (ng/L)	IgG (*μ*g/L)	IgM (mg/L)
Control (30)^2^	213.20 ± 6.79^1b^	338.73 ± 18.45^b^	263.38 ± 4.58^b^	48.53 ± 3.42^a^
Group SE (17)	237.32 ± 10.80^ab^	404.19 ± 26.01^a^	263.08 ± 5.29^b^	44.71 ± 5.44^a^
Group SP (18)	232.55 ± 10.91^ab^	357.98 ± 32.64^ab^	282.91 ± 8.46^a^	56.63 ± 6.49^a^
Poly(I:C) (20)	239.24 ± 11.83^a^	383.04 ± 19.32^ab^	264.70 ± 5.35^b^	45.41 ± 4.27^a^

^1^Mean ± standard error; ^2^number of individuals in groups. In a given column, the same letter indicates that there is no significant difference (*P*  value ≤ 0.05).

**Table:** (a)

Probe name	*P* value	|FC|	Group SP (raw_data)	Control (raw_data)	Gene symbol
A_87_P135073	0.0273	2.6414	38479.96	21784.10	RANGAP1
A_87_P068266	0.0299	2.7656	4204.96	2270.40	OGCHI
A_87_P063991	0.0130	2.4764	2527.43	1473.75	AIM1L
A_87_P158638	0.0320	2.9393	1893.88	972.62	SLCO1B3
A_87_P034912	0.0135	2.4772	1504.71	855.34	ITM2C
A_87_P307413	0.0331	2.5736	247.74	141.09	VAV2
A_87_P152483	0.0188	3.7106	244.17	96.53	FREM1

**Table:** (b)

Probe name	*P* value	|FC|	Group SP (raw_data)	Control (raw_data)	Gene symbol
A_87_P263263	0.0337	3.7427	15599.45	72900.14	RSFR
A_87_P009488	0.0477	2.6393	1483.44	4893.00	MERTK
A_87_P021714	0.0162	2.3141	467.72	1469.43	RAB32
A_87_P155463	0.0420	2.1985	1043.95	3037.73	TPRN
A_87_P106828	0.0105	2.1289	685.27	1982.21	TMEM140
A_87_P036207	0.0202	2.0998	3873.45	10873.39	C3AR1

**Table:** (a)

Probe name	*P* value	|FC|	Group SP (raw_data)	Control (raw_data)	Gene symbol
A_87_P017067	0.014833	2.241353	27158.97	10506.86	CLEC3B
A_87_P055986	0.026444	2.048135	9172.174	3898.472	PDLIM3
A_87_P110393	0.044918	2.134904	8211.222	3363.067	FHL2
A_87_P193418	0.046498	2.219647	7528.244	2944.842	FHL2
A_87_P213848	0.026094	2.937232	6073.972	1757.178	LOC423423
A_87_P265788	0.047425	2.028906	5994.927	2552.497	BAG2
A_87_P055721	0.004181	2.57088	5538.785	1861.226	HOXD11
A_87_P009294	0.004336	2.365375	3699.251	1356.083	HOXD11
A_87_P080001	0.006183	2.206574	2488.212	976.5921	CISD1
A_87_P245708	0.033453	2.915283	2122.973	633.1538	ANLN
A_87_P034042	0.040937	4.771468	2106.639	398.8331	ACTC1
A_87_P051766	0.046243	2.132916	1784.825	733.5394	LOC418424
A_87_P151068	0.008562	2.248188	1701.529	656.2959	MAMDC2
A_87_P037492	0.021	2.189082	1638.75	647.6197	PDLIM3
A_87_P009048	0.026677	2.046262	1590.921	674.5408	CENPF
A_87_P085056	0.046133	2.137321	1406.97	564.0237	SCG5
A_87_P092561	0.009984	2.567488	1324.834	446.0624	MAMDC2
A_87_P152008	0.004968	2.519559	775.9677	266.9718	KLHDC8A
A_87_P027905	0.019624	2.050455	704.9199	299.1262	COMP
A_87_P054746	0.021351	2.122426	545.0016	223.2091	CENPF
A_87_P102401	0.003087	2.933887	352.884	104.1954	ANLN
A_87_P236533	0.046516	7.077659	318.3763	37.00138	C20H20orf85
A_87_P104513	0.002662	3.046689	315.2515	89.91791	MYBL1
A_87_P179618	0.018637	3.150793	305.2546	84.68122	LOC768920
A_87_P319922	0.024433	3.271542	299.3379	78.5472	LOC768920
A_87_P022300	0.046964	2.042768	291.6057	122.5599	RIC3
A_87_P145383	0.039066	2.329995	282.5269	105.3242	SLIT1
A_87_P104908	0.009377	2.041087	208.8738	88.86916	OSR2
A_87_P021436	0.04428	2.539008	183.9284	63.00039	TUB
A_87_P037697	0.043356	3.221175	163.8235	45.27623	COL20A1
A_87_P296773	0.039883	2.340923	156.9485	58.23907	ALDH1L2
A_87_P037881	0.00596	3.139463	155.8588	43.17721	SOUL
A_87_P053201	0.026585	2.044309	141.0832	59.69648	SYNPR
A_87_P037934	0.034472	8.460807	139.0743	15.11028	GDF5
A_87_P056771	0.021345	2.403871	131.1553	47.09236	GLP1R
A_87_P106548	0.033798	2.003954	122.4247	53.13174	TRHDE
A_87_P101681	0.006572	2.674664	117.2865	37.99509	DGKB
A_87_P054406	0.029114	2.063656	110.2237	46.68424	OXTR

**Table:** (b)

Probe name	*P* value	|FC|	Group SP (raw_data)	Control (raw_data)	Gene symbol
A_87_P081341	0.008753	2.100018	5147.149	9383.597	LOC423942
A_87_P124573	0.018046	2.063123	4147.323	7464.053	MOV10
A_87_P034814	0.007111	2.76483	2803.302	6681.683	IFIT5
A_87_P081336	0.008268	2.142918	2141.293	3960.148	LOC423942
A_87_P275528	0.028744	2.018683	1590.584	2771.403	PSAP
A_87_P094106	0.046616	2.009193	998.6245	1720.768	GPR112
A_87_P315787	0.01107	2.017383	404.4446	704.7305	PARP14
A_87_P129690	0.012764	2.245596	218.1902	424.9018	CATHL3
A_87_P263168	0.027271	2.157093	171.706	317.8527	PLBD1
A_87_P018024	0.028947	12.86841	147.5546	1823.653	CD72
A_87_P022808	0.042314	2.469146	122.4936	258.4686	MOG
A_87_P016668	0.039132	2.424182	118.4504	252.0765	CILP

**Table 5:** Four annotated DEGs found in the spleen in all three treatment groups.

ID	Gene name	Gene symbol	Regulation
A_87_P014094	RNA binding motif protein 16	RBM16	Up
A_87_P022310	fumarylacetoacetate hydrolase (fumarylacetoacetase)	FAH	Up
A_87_P037218	SRY (sex determining region Y)-box 5	SOX5	Up
A_87_P150753	RNA binding protein, fox-1 homolog (C. elegans)2	RBM9	Up

**Table 6:** Four annotated DEGs found in the caecum of all three treatment groups.

ID	Gene name	Gene symbol	Regulation
A_87_P037881	SOUL protein	SOUL	Up
A_87_P012535	ficolin (collagen/fibrinogen domain containing lectin) 2 (hucolin)	FCN2	Down
A_87_P102401	anillin, actin binding protein	ANLN	Up
A_87_P056446	Gallus gallus acyl-CoA synthetase long-chain family member 1 (ACSL1)	ACSL1	Down

**Table 7:** Results of GO biology process enrichment analysis of DEGs in the spleen of group SP (*P* value ≤ 0.05).

Term	Count	*P* value
Hemopoiesis	7	8.61*E* − 04
Hemopoietic or lymphoid organ development	7	0.001563043
Immune system development	7	0.001998123
Cell activation	6	0.003897752
Lymphocyte differentiation	4	0.015888222
Positive regulation of cell proliferation	6	0.025606698
Leukocyte differentiation	4	0.030403458
Positive regulation of mononuclear cell proliferation	3	0.037956297
Positive regulation of leukocyte proliferation	3	0.037956297
Positive regulation of lymphocyte proliferation	3	0.037956297
Lymphocyte activation	4	0.046396956
Regulation of mononuclear cell proliferation	3	0.046703876
Regulation of leukocyte proliferation	3	0.046703876
Regulation of lymphocyte proliferation	3	0.046703876
T-cell differentiation	3	0.05611028

**Table 8:** Results of GO biology process enrichment analysis of DEGs in the caecum of group SP (*P* value ≤ 0.05).

Term	Count	%	*P* value
Enrichment score: 2.9222	****	****	****
Response to bacteria	5	5.154639	3.43*E* − 04
Defense response to bacteria	4	4.123711	0.001003
Defense response	5	5.154639	0.004978
Enrichment score: 2.1436			
Circulatory system process	4	4.123711	0.005254
Blood circulation	4	4.123711	0.005254
Regulation of blood pressure	3	3.092784	0.013437
Enrichment score: 1.3467			
Positive regulation of I-kappaB kinase/NF-kappaB cascade	3	3.092784	0.012329
Regulation of I-kappaB kinase/NF-kappaB cascade	3	3.092784	0.013437
Positive regulation of protein kinase cascade	3	3.092784	0.039886

**Table 9:** DEGs involved in immune process in the spleen of group SP.

Probe IDs	Gene names	Regulation
A_87_P009188	paired box gene 6	Down
A_87_P037253	cholecystokinin B receptor	Up
A_87_P037851	interleukin 15	Down
A_87_P008861	CD28 molecule	Down
A_87_P035516	interleukin 7	Up
A_87_P009462	interferon regulatory factor 8	Down
A_87_P021863	Notch homolog 1, translocation-associated (Drosophila)	Up
A_87_P013809	general transcription factor IIH, polypeptide 4, 52 kDa	Up
A_87_P038093	mutS homolog 2, colon cancer, nonpolyposis type 1 (*E. coli*)	Up
A_87_P037741	similar to twisted gastrulation; twisted gastrulation homolog 1 (Drosophila)	Up
A_87_P013467	homeobox A9	Up

**Table 10:** DEGs involved in immune process in the caecum of group SP.

Probe IDs	Gene name	Regulation
A_87_P035105	Gal 7	Down
A_87_P024004	gallinacin 1	Down
A_87_P009700	myeloid antimicrobial peptide 27	Down
A_87_P036472	receptor-interacting serine-threonine kinase 2	Down
A_87_P038151	toll-like receptor 2	Down
A_87_P037776	5-Hydroxytryptamine (serotonin) receptor 2B	Up
A_87_P035041	adiponectin, C1Q and collagen domain containing	Up
A_87_P036472	receptor-interacting serine-threonine kinase 2	Down

**Table 11:** qRT-PCR results of five DEGs selected from microarray data. (*P* value ≤ 0.05).

Gene symbols	|FC| of microarray data	Expression trend of microarray data	Relative expression level of qRT-PCR	Significance (*P* value ≤ 0.05)	Expression trend of qRT-PCR
RSFR	0.27	Down	0.00	0.04	Down
IL15	0.54	Down	4.51	0.10	No difference
VPREB3	7.55	Up	535.57	0.00	Up
CD72	0.08	Down	0.00	0.02	Down
GAL1	0.02	Down	0.00	0.00	Down

## References

[1] Suzuki S (1994). Pathogenicity of *Salmonella enteritidis* in poultry. *International Journal of Food Microbiology*.

[2] Barrow PA, Huggins MB, Lovell MA, Simpson JM (1987). Observations on the pathogenesis of experimental *Salmonella typhimurium* infection in chickens. *Research in Veterinary Science*.

[3] Tanimura N, Saitoh S, Matsumoto F, Akashi-Takamura S, Miyake K (2008). Roles for LPS-dependent interaction and relocation of TLR4 and TRAM in TRIF-signaling. *Biochemical and Biophysical Research Communications*.

[4] Li P, Xia P, Wen J (2010). Up-regulation of the MyD88-dependent pathway of TLR signaling in spleen and caecum of young chickens infected with *Salmonella serovar Pullorum*. *Veterinary Microbiology*.

[5] Ramasamy KT, Verma P, Reddy MR (2012). Differential gene expression of antimicrobial peptides *β* defensins in the gastrointestinal tract of *Salmonella serovar Pullorum* infected broiler chickens. *Veterinary Research Communications*.

[6] Withanage GSK, Kaiser P, Wigley P (2004). Rapid expression of chemokines and proinflammatory cytokines in newly hatched chickens infected with *Salmonella enterica serovar typhimurium*. *Infection and Immunity*.

[7] Withanage GSK, Wigley P, Kaiser P (2005). Cytokine and chemokine responses associated with clearance of a primary *Salmonella enterica serovar typhimurium* infection in the chicken and in protective immunity to rechallenge. *Infection and Immunity*.

[8] van Hemert S, Hoekman AJW, Smits MA, Rebel JMJ (2006). Gene expression responses to a *Salmonella* infection in the chicken intestine differ between lines. *Veterinary Immunology and Immunopathology*.

[9] van Hemert S, Hoekman AJW, Smits MA, Rebel JMJ (2006). Early host gene expression responses to a *Salmonella* infection in the intestine of chickens with different genetic background examined with cDNA and oligonucleotide microarrays. *Comparative Biochemistry and Physiology D*.

[10] van Hemert S, Hoekman AJW, Smits MA, Rebel JMJ (2007). Immunological and gene expression responses to a *Salmonella* infection in the chicken intestine. *Veterinary Research*.

[11] Schokker D, de Koning D-J, Rebel JMJ, Smits MA (2011). Shift in chicken intestinal gene association networks after infection with *Salmonella*. *Comparative Biochemistry and Physiology D*.

[12] Lee CC, Wu CC, Lin TL (2012). Characterization of chicken melanoma differentiation-associated gene 5 (MDA5) from alternative translation initiation. *Comparative Immunology, Microbiology & Infectious Diseases*.

[13] Lian L, Ciraci C, Chang G, Hu J, Lamont SJ (2012). NLRC5 knockdown in chicken macrophages alters response to LPS and poly (I:C) stimulation. *BMC Veterinary Research*.

[14] Oshiumi H, Matsumoto M, Funami K, Akazawa T, Seya T (2003). TICAM-1, an adaptor molecule that participates in Toll-like receptor 3-mediated interferon-*β* induction. *Nature Immunology*.

[15] Chunmei C, Yao S, Maoyan T, Jinhua L, Rongchun Z, Cheng J (2006). Effects of levels of dietary protein and lysine on growth and meat quality of AA broilers. *Journal of China Agricultural University*.

[16] Gu Y, Xia Y, Chen Q, Sha W, Dai H, Zhu J (2009). Affection of fattening in Growth stage of the Langshan Breed. *Journal of Yangtze University*.

[17] Xinsheng W, Guohong C, Kuanwei C, Kehua W, Hong C (1998). Comparison on histologic characteristics of muscle and muscle quality in Chinese native chickens. *Journal of Yangzhou University*.

[18] Te Pas MF, Hulsegge I, Schokker D (2012). Meta-analysis of chicken—*Salmonella* infection experiments. *BMC Genomics*.

[19] Schokker D, Peters THF, Hoekman AJW, Rebel JMJ, Smits MA (2012). Differences in the early response of hatchlings of different chicken breeding lines to *Salmonella enterica* serovar Enteritidis infection. *Poultry Science*.

[20] Bolstad BM, Irizarry RA, Åstrand M, Speed TP (2003). A comparison of normalization methods for high density oligonucleotide array data based on variance and bias. *Bioinformatics*.

[21] Huang DW, Sherman BT, Lempicki RA (2009). Systematic and integrative analysis of large gene lists using DAVID bioinformatics resources. *Nature Protocols*.

[22] Pfaffl MW (2001). A new mathematical model for relative quantification in real-time RT-PCR. *Nucleic Acids Research*.

[23] Pfaffl MW, Horgan GW, Dempfle L (2002). Relative expression software tool (REST) for group-wise comparison and statistical analysis of relative expression results in real-time PCR. *Nucleic acids research*.

[24] Boettcher JP, Kirchner M, Churin Y (2010). Tyrosine-phosphorylated caveolin-1 blocks bacterial uptake by inducing Vav2-RhoA-mediated cytoskeletal rearrangements. *PLoS Biology*.

[25] Nitto T, Dyer KD, Czapiga M, Rosenberg HF (2006). Evolution and function of leukocyte RNase A ribonucleases of the avian species, Gallus gallus. *Journal of Biological Chemistry*.

[26] Ostergaard E, Duno M, Batbayli M, Vilhelmsen K, Rosenberg T (2011). A novel MERTK deletion is a common founder mutation in the faroe islands and is responsible for a high proportion of retinitis pigmentosa cases. *Molecular Vision*.

[27] Linger RMA, Keating AK, Earp HS, Graham DK (2008). TAM receptor tyrosine kinases: biologic functions, signaling, and potential therapeutic targeting in human cancer. *Advances in Cancer Research*.

[28] Wang C, Liu Z, Huang X (2012). Rab32 is important for autophagy and lipid storage in drosophila. *PLoS ONE*.

[29] Bultema JJ, Ambrosio AL, Burek CL, di Pietro SM (2012). BLOC-2, AP-, and AP-1 proteins function in concert with Rab38 and Rab32 proteins to mediate protein trafficking to lysosome-related organelles. *The Journal of Biological Chemistry*.

[30] Zhang FR, Liu H, Chen SM (2011). Identification of two new loci at IL23R and RAB32 that influence susceptibility to leprosy. *Nature Genetics*.

[31] Li Y, Pohl E, Boulouiz R (2010). Mutations in TPRN cause a progressive form of autosomal-recessive nonsyndromic hearing loss. *American Journal of Human Genetics*.

[32] Shimizu N, Noda S, Katayama K, Ichikawa H, Kodama H, Miyoshi H (2008). Identification of genes potentially involved in supporting hematopoietic stem cell activity of stromal cell line MC3T3-G2/PA6. *International Journal of Hematology*.

[33] Rosnet O, Blanco-Betancourt C, Grivel K, Richter K, Schiff C (2004). Binding of free immunoglobulin light chains to VpreB3 inhibits their maturation and secretion in chicken B cells. *Journal of Biological Chemistry*.

[34] Wong CH, Mak GWY, Li MS, Tsui SKW (2012). The LIM-only protein FHL2 regulates interleukin-6 expression through p38 MAPK mediated NF-kappa B pathway in muscle cells. *Cytokine*.

[35] Ohsawa N, Koebis M, Suo S, Nishino I, Ishiura S (2011). Alternative splicing of PDLIM3/ALP, for *α*-actinin-associated LIM protein 3, is aberrant in persons with myotonic dystrophy. *Biochemical and Biophysical Research Communications*.

[36] Vanderven HA, Petkau K, Ryan-Jean KEE, Aldridge JR, Webster RG, Magor KE (2012). Avian influenza rapidly induces antiviral genes in duck lung and intestine. *Molecular Immunology*.

[37] Øvstebø R, Olstad OK, Brusletto B (2008). Identification of genes particularly sensitive to lipopolysaccharide (LPS) in human monocytes induced by wild-type versus LPS-deficient Neisseria meningitidis strains. *Infection and Immunity*.

[38] Daffis S, Szretter KJ, Schriewer J (2010). 2′-O methylation of the viral mRNA cap evades host restriction by IFIT family members. *Nature*.

[39] Alcón VL, Luther C, Balce D, Takei F (2009). B-cell co-receptor CD72 is expressed on NK cells and inhibits IFN-*γ* production but not cytotoxicity. *European Journal of Immunology*.

[40] Miller MM, Goto R, Young S, Chirivella J, Hawke D, Miyada CG (1991). Immunoglobulin variable-region-like domains of diverse sequence within the major histocompatibility complex of the chicken. *Proceedings of the National Academy of Sciences of the United States of America*.

[41] Ambrosi E, Capaldi S, Bovi M, Saccomani G, Perduca M, Monaco HL (2011). Structural changes in the BH3 domain of SOUL protein upon interaction with the anti-apoptotic protein Bcl-xL. *Biochemical Journal*.

[42] Garred P, Honoré C, Ma YJ (2009). The genetics of ficolins. *Journal of Innate Immunity*.

[43] Clements M, Eriksson S, Tezcan-Merdol D, Hinton JCD, Rhen M (2001). Virulence gene regulation in *Salmonella enterica*. *Annals of Medicine*.

[44] Vervelde L, Reemers SS, van Haarlem DA (2013). Chicken dendritic cells are susceptible to highly pathogenic avian influenza viruses which induce strong cytokine responses. *Developmental & Comparative Immunology*.

[45] Schoenberger SP, Pulendran B, Katsikis PD (2012). 4th aegean conference on the crossroads between innate and adaptive immunity. *Nature Immunology*.

[46] Setta AM, Barrow PA, Kaiser P, Jones MA (2012). Early immune dynamics following infection with *Salmonella enterica* serovars Enteritidis, Infantis, Pullorum and Gallinarum: cytokine and chemokine gene expression profile and cellular changes of chicken cecal tonsils. *Comparative Immunology, Microbiology and Infectious Diseases*.

[47] Zola H (2006). Medical applications of leukocyte surface molecules—The CD molecules. *Molecular Medicine*.

[48] Lillehoj HS, Min W, Choi KD (2001). Molecular, cellular, and functional characterization of chicken cytokines homologous to mammalian IL-15 and IL-2. *Veterinary Immunology and Immunopathology*.

[49] Johnson K, Chaumeil J, Micsinai M (2012). IL-7 functionally segregates the Pro-B cell stage by regulating transcription of recombination mediators across cell cycle. *Journal of Immunology*.

[50] Yang DF, Thangaraju M, Greeneltch K (2007). Repression of IFN regulatory factor 8 by DNA methylation is a molecular determinant of apoptotic resistance and metastatic phenotype in metastatic tumor cells. *Cancer Research*.

[51] Yang DF, Wang S, Brooks C (2009). IFN regulatory factor 8 sensitizes soft tissue sarcoma cells to death receptor-initiated apoptosis via repression of FLICE-like protein expression. *Cancer Research*.

[52] Osipo C, Golde TE, Osborne BA, Miele LA (2008). Off the beaten pathway: the complex cross talk between Notch and NF-*κ*B. *Laboratory Investigation*.

[53] Wheaton S, Lambourne MD, Sarson AJ, Brisbin JT, Mayameei A, Sharif S (2007). Molecular cloning and expression analysis of chicken MyD88 and TRIF genes. *DNA Sequence*.

[54] Díez JJ, Iglesias P (2003). The role of the novel adipocyte-derived hormone adiponectin in human disease. *European Journal of Endocrinology*.

[55] Gibson MS, Fife M, Bird S, Salmon N, Kaiser P (2012). Identification, cloning, and functional characterization of the IL-1 receptor antagonist in the chicken reveal important differences between the chicken and mammals. *The Journal of Immunology*.

[56] Karpala AJ, Stewart C, McKay J, Lowenthal JW, Bean AGD (2011). Characterization of chicken Mda5 activity: regulation of IFN-*β* in the absence of RIG-I functionality. *Journal of Immunology*.

[57] Keestra AM, de Zoete MR, Bouwman LI, van Putten JPM (2010). Chicken TLR21 is an innate CpG DNA receptor distinct from mammalian TLR9. *Journal of Immunology*.

