# Supplementary material for: Identification of Key Genes in the Response to Salmonella enterica Enteritidis, Salmonella enterica Pullorum, and Poly(I:C) in Chicken Spleen and Caecum
Source: Biomed Res Int. 2014 Feb 23;2014:154946. doi: 10.1155/2014/154946 (PMC3950952; doi:10.1155/2014/154946)
Supplement: Supplementary file 1 — Table S1-1: Upregulation of differentially expressed genes in the spleen in group SP with FC ≥ 2 and Signal Density of Raw Data ≥ 100. Table S1-2: Downregulation of differentially expressed genes in the spleen in group SP with FC ≥ 2 and Signal Density of Raw Data ≥ 100. Table S2-1: Upregulation of differentially expressed genes in the spleen in group SE with FC ≥ 2 and Signal Density of Raw Data ≥ 100. Table S2-2: Downregulation of differentially expressed genes in the spleen in group SE with FC ≥ 2 and Signal Density of Raw Data ≥ 100. Table S3-1: Upregulation of differentially expressed genes in the spleen in group Poly(I:C) with FC ≥ 2 and Signal Density of Raw Data ≥ 100. Table S3-2: Downregulation of differentially expressed genes in the spleen in group Poly(I:C) with FC ≥ 2 and Signal Density of Raw Data ≥ 100. Table S4-1: Upregulation of differentially expressed genes in the caecum in group SP with FC ≥ 2 and Signal Density of Raw Data ≥ 100. Table S4-2: Downregulation of differentially expressed genes in the caecum in group SP with FC ≥ 2 and Signal Density of Raw Data ≥ 100. Table S5-1: Upregulation of differentially expressed genes in the caecum in group SE with FC ≥ 2 and Signal Density of Raw Data ≥ 100. Table S5-2: Downregulation of differentially expressed genes in the caecum in group SE with FC ≥ 2 and Signal Density of Raw Data ≥ 100. Table S6-1: Upregulation of differentially expressed genes in the caecum in group Poly(I:C) with FC ≥ 2 and Signal Density of Raw Data ≥ 100. Table S6-2: Downregulation of differentially expressed genes in the caecum in group Poly(I:C) with FC ≥ 2 and Signal Density of Raw Data ≥ 100. Table S7: qRT-PCR primers for 5 target genes. Table S8: Statistical description of the difference in the expression of genes in each group in the spleen. Table S9: Statistical description of the difference in the expression of genes in each group in the caecum. [file 154946.f1.pdf]

Identification of key genes in the response to Salmonella enteritidis, Salmonella pullorum and Poly(I:C) in chicken spleen and caecum

Teng Ma[a], Guobin Chang[a]*, Rong Chen[a], Zhongwei Sheng[a], Aiqin Dai[a], Fei Zhai[a], Jianchao Li[a], Mingxiu Xia[a], Dengke Hua[a], Lu Xu[a], Hongzhi Wang[a], Jing Chen[a], Lu Liu[a], Guohong Chen[a]*

[a]Animal genetic resources laboratory, College of Animal Science and Technology, Yangzhou University, 88 South of University Ave., Yangzhou, Jiangsu, 225009, P. R. China

*Correspondence to:

Guobin Chang, Ph.D.

Animal genetic resources laboratory, College of Animal Science and Technology, Yangzhou University, 88 South of University Ave., Yangzhou, Jiangsu, 225009, P. R. China

Tel. +86-514-87979034

E-mail: passioncgb@163.com

Or Guohong Chen, Ph.D.

Animal genetic resources laboratory, College of Animal Science and Technology, Yangzhou University, 88 South of University Ave., Yangzhou, Jiangsu, 225009, P. R. China

E-mail: ghchen@yzu.edu.cn

Supplementary Tables

Table S1-1 Upregulation of differentially expressed genes in the spleen in group SP with FC≥2 and Signal Density of Raw Data ≥100.

Table S1-2 Downregulation of differentially expressed genes in the spleen in group SP with FC≥2 and Signal Density of Raw Data ≥100.

Table S2-1 Upregulation of differentially expressed genes in the spleen in group SE with FC≥2 and Signal Density of Raw Data ≥100.

Table S2-2 Downregulation of differentially expressed genes in the spleen in group SE with FC≥2 and Signal Density of Raw Data ≥100.

Table S3-1 Upregulation of differentially expressed genes in the spleen in group Poly(I:C) with FC≥2 and Signal Density of Raw Data ≥100.

Table S3-2 Downregulation of differentially expressed genes in the spleen in group Poly(I:C) with FC≥2 and Signal Density of Raw Data ≥100.

Table S4-1 Upregulation of differentially expressed genes in the caecum in group SP with FC≥2 and Signal Density of Raw Data ≥100.

Table S4-2 Downregulation of differentially expressed genes in the caecum in group SP with FC≥2 and Signal Density of Raw Data ≥100.

Table S5-1 Upregulation of differentially expressed genes in the caecum in group SE with FC≥2 and Signal Density of Raw Data ≥100.

Table S5-2 Downregulation of differentially expressed genes in the caecum in group SE with FC≥2 and Signal Density of Raw Data ≥100.

Table S6-1 Upregulation of differentially expressed genes in the caecum in group Poly(I:C) with FC≥2 and Signal Density of Raw Data ≥100.

Table S6-2 Downregulation of differentially expressed genes in the caecum in group Poly(I:C) with FC≥2 and Signal Density of Raw Data ≥100.

Table S7 qRT-PCR primers for 5 target genes.

Table S8 Statistical description of the difference in the expression of genes in each group in the spleen.

Table S9 Statistical description of the difference in the expression of genes in each group in the caecum.

Table S1-1 Upregulation of differentially expressed genes in the spleen in group SP with FC≥2 and Signal Density of Raw Data ≥100.

ProbeName	p-value	|FC|	Regulation	GROUP SP(Raw_data)	CONTROL_(Raw_data)	GeneSymbol
A_87_P135073	0.0273	2.6414	up	38479.96	21784.10	RANGAP1
A_87_P303693	0.0484	2.0890	up	5867.15	4189.91	Unknown
A_87_P108768	0.0055	3.0862	up	4046.30	1769.06	Unknown
A_87_P068266	0.0299	2.7656	up	4204.96	2270.40	OGCHI
A_87_P063991	0.0130	2.4764	up	2527.43	1473.75	AIM1L
A_87_P016314	0.0130	3.1166	up	2407.87	1143.18	Unknown
A_87_P013273	0.0009	2.8880	up	2099.30	1030.90	Unknown
A_87_P023770	0.0233	2.6145	up	2052.43	1168.90	Unknown
A_87_P158638	0.0320	2.9393	up	1893.88	972.62	SLCO1B3
A_87_P034912	0.0135	2.4772	up	1504.71	855.34	ITM2C
A_87_P309777	0.0403	3.1039	up	415.25	200.11	Unknown
A_87_P177233	0.0115	2.9469	up	382.11	184.15	Unknown
A_87_P011704	0.0478	8.1813	up	443.44	86.93	Unknown
A_87_P094056	0.0015	2.7755	up	285.54	144.89	Unknown
A_87_P307413	0.0331	2.5736	up	247.74	141.09	VAV2
A_87_P152483	0.0188	3.7106	up	244.17	96.53	FREM1

Table S1-2 Downregulation of differentially expressed genes in the spleen in group SP with FC≥2 and Signal Density of Raw Data ≥100.

ProbeName	p-value	|FC|	Regulation	GROUP SP(Raw_data)	CONTROL_(Raw_data)	GeneSymbol
A_87_P263263	0.0337	3.7427	down	15599.45	72900.14	RSFR
A_87_P024462	0.0428	2.6697	down	2296.36	9102.05	Unknown
A_87_P009488	0.0477	2.6393	down	1483.44	4893.00	MERTK
A_87_P002428	0.0352	2.5310	down	1403.64	5026.98	Unknown
A_87_P021714	0.0162	2.3141	down	467.72	1469.43	RAB32
A_87_P311142	0.0385	2.3097	down	8515.84	25377.76	Unknown
A_87_P155463	0.0420	2.1985	down	1043.95	3037.73	TPRN
A_87_P106828	0.0105	2.1289	down	685.27	1982.21	TMEM140
A_87_P119743	0.0104	2.1078	down	1086.69	3119.70	Unknown
A_87_P036207	0.0202	2.0998	down	3873.45	10873.39	C3AR1

Table S2-1 Upregulation of differentially expressed genes in the spleen in group SE with FC≥ 2 and Signal Density of Raw Data ≥100.

ProbeName	p-value	|FC|	Regulation	GROUP SE(RAW_DATA)	CONTROL(RAW_DATA)	GeneSymbol
A_87_P116658	0.0261	2.8863	up	43378.40	18276.59	
A_87_P017937	0.0113	2.9774	up	29694.70	12650.07	
A_87_P300758	0.0031	2.1611	up	16723.76	9607.29	ATP1A1
A_87_P054071	0.0128	2.1016	up	15168.78	8858.81	CIRBP
A_87_P009358	0.0377	2.0595	up	10933.91	6790.29	TGFB3
A_87_P128243	0.0461	2.0723	up	10872.15	6718.23	TGFB3
A_87_P009201	0.0443	2.3729	up	7060.30	3823.04	CTSD
A_87_P009546	0.0002	2.1524	up	5226.66	3026.52	ATP1A1
A_87_P015764	0.0029	3.4883	up	4564.30	1611.47	
A_87_P037826	0.0268	2.1204	up	4242.24	2488.16	SPRY1
A_87_P057585	0.0101	2.0849	up	3599.72	2167.43	ACTA2
A_87_P006702	0.0053	2.4163	up	3576.10	1867.09	
A_87_P009800	0.0136	2.1009	up	3359.74	1945.28	PTRF
A_87_P012337	0.0114	2.3253	up	2830.29	1480.60	MAFF
A_87_P093886	0.0005	2.0960	up	2482.78	1472.80	LOC422179
A_87_P113483	0.0113	2.0754	up	1548.61	906.54	TFPI
A_87_P063651	0.0259	2.2563	up	1322.75	733.79	G0S2
A_87_P054871	0.0190	2.0980	up	1190.79	720.97	RGS4
A_87_P126458	0.0308	2.3923	up	1078.64	532.25	
A_87_P021537	0.0092	2.1109	up	1054.81	630.69	RGS4
A_87_P011006	0.0298	2.1782	up	751.33	440.28	ENOPH1
A_87_P193858	0.0254	2.4060	up	748.50	392.53	TAGLN
A_87_P007472	0.0155	2.2673	up	591.55	331.69	
A_87_P023735	0.0371	2.5534	up	564.49	284.07	CA5B
A_87_P114813	0.0116	2.1780	up	560.23	312.30	TFPI
A_87_P053141	0.0275	2.4644	up	559.32	288.93	TAGLN
A_87_P031921	0.0052	2.0564	up	521.13	318.24	SEC14L2
A_87_P017770	0.0096	2.7064	up	517.53	230.91	CXCR7
A_87_P290988	0.0069	2.6124	up	484.74	231.39	COL6A3
A_87_P037837	0.0074	2.1143	up	476.64	276.35	PDLIM4
A_87_P196268	0.0299	2.1667	up	471.38	272.22	LPL
A_87_P116118	0.0021	2.8828	up	465.19	199.74	
A_87_P021557	0.0034	2.1640	up	419.15	238.16	TFPI
A_87_P073586	0.0186	2.1313	up	373.87	211.34	
A_87_P056971	0.0383	2.2831	up	370.12	194.49	LPL
A_87_P133583	0.0314	2.4050	up	321.89	170.86	TAGLN
A_87_P051101	0.0216	2.9783	up	302.98	120.78	TNIP2
A_87_P023932	0.0369	2.4445	up	271.35	130.52	
A_87_P084991	0.0035	2.8191	up	268.47	119.95	
A_87_P079216	0.0163	2.3986	up	238.48	121.44	ST3GAL3

A_87_P124693	0.0198	2.3824	up	230.19	117.59	
A_87_P013987	0.0337	2.6960	up	225.49	107.47	
A_87_P229933	0.0059	2.4065	up	215.28	112.98	LOC416032
A_87_P012660	0.0497	2.7031	up	193.91	84.48	
A_87_P008994	0.0202	2.3507	up	192.92	104.36	KCNJ8
A_87_P078396	0.0008	2.8549	up	186.13	80.52	REG4
A_87_P102461	0.0075	2.2448	up	171.79	95.92	LOC776557
A_87_P024344	0.0431	2.9563	up	154.14	63.08	CREB3L3
A_87_P069301	0.0162	2.6927	up	124.60	55.81	WSCD2
A_87_P109288	0.0265	2.0863	up	119.30	69.54	CXADR

Table S2-2 Downregulation of differentially expressed genes in the spleen in group SE with FC≥2 and Signal Density of Raw Data ≥100.

ProbeName	p-value	|FC|	Regulation	GROUP SE(RAW_DATA)	CONTROL(RAW_DATA)	GeneSymbol
A_87_P054276	0.0447	2.0652	down	16448.06	43485.36	SEPP1
A_87_P014410	0.0324	2.1137	down	559.10	1503.89	Unknown
A_87_P013310	0.0011	2.2054	down	487.68	1341.91	NFYA
A_87_P105043	0.0280	7.7681	down	472.24	4752.52	RIMS2
A_87_P018662	0.0057	2.0215	down	384.47	969.83	LOC417740
A_87_P094796	0.0299	2.3384	down	281.53	841.93	TMEM192
A_87_P024624	0.0153	3.2553	down	257.03	1064.79	Unknown
A_87_P013373	0.0030	2.6872	down	204.34	681.21	Unknown
A_87_P017706	0.0309	3.6033	down	179.42	834.44	MPZL3
A_87_P111278	0.0150	2.2338	down	178.27	483.12	LOC418927
A_87_P016037	0.0384	2.1368	down	152.10	384.82	Unknown
A_87_P014829	0.0356	2.1536	down	132.27	349.11	Unknown
A_87_P011380	0.0345	2.0770	down	126.28	330.35	Unknown
A_87_P026669	0.0100	5.3427	down	117.76	803.74	LOC771876
A_87_P114948	0.0330	4.2311	down	110.76	616.50	C6H10orf71
A_87_P065136	0.0210	3.4744	down	106.61	475.74	MPZL3

Table S3-1 Upregulation of differentially expressed genes in the spleen in group Poly(I:C) with FC≥2 and Signal Density of Raw Data ≥100.

ProbeName	p-value	|FC|	Regulation	GROUP POLY(I:C)(RAW_DATA)	CONTROL(RAW_DATA)	GeneSymbol
A_87_P057585	0.024516	2.105607	up	2927.151	2167.429	ACTA2
A_87_P012813	0.045663	2.02824	up	2914.729	2273.507	UTS2D
A_87_P107513	0.042305	2.025086	up	2386.644	1819.623	CKAP4
A_87_P018301	0.019989	2.002368	up	2140.571	1662.255	
A_87_P069601	0.019984	7.549002	up	2029.871	424.2184	VPREB3
A_87_P121898	0.024996	4.438685	up	1781.27	608.2567	
A_87_P249113	0.038541	2.178563	up	1417.42	1025.3	JARID1A
A_87_P053436	0.005433	2.1703	up	1417.062	1020.713	MIS12
A_87_P115598	0.017036	2.358013	up	1325.665	872.1884	COL4A1
A_87_P014094	0.015296	2.444043	up	1167.924	746.7803	RBM16
A_87_P324877	0.003539	2.401552	up	1150.94	749.7606	
A_87_P055971	0.002829	2.313432	up	1037.796	699.4371	LOC427896
A_87_P014007	0.017124	2.305064	up	876.8105	590.411	
A_87_P175073	0.003958	2.146015	up	874.8078	636.5988	11-Sep
A_87_P104643	0.040575	2.018552	up	761.3413	593.6819	C8orf70
A_87_P022970	0.044222	2.289751	up	693.0373	478.6051	AMOT
A_87_P149298	0.040277	2.00665	up	650.3829	504.2125	FIBIN
A_87_P124003	0.026831	2.047612	up	634.8118	485.4089	MAP7D1
A_87_P314777	0.005215	2.462464	up	628.238	399.3048	
A_87_P096116	0.049756	2.301085	up	565.8392	376.5663	RASL11B
A_87_P188610	0.01939	3.557655	up	522.7618	229.4975	
A_87_P015444	0.025249	2.494155	up	512.5837	323.0428	CNST
A_87_P081041	0.023942	2.596892	up	500.9981	299.9647	C6H10orf26
A_87_P022659	0.01852	3.325711	up	470.723	222.9621	
A_87_P086317	0.039026	3.464577	up	468.7569	216.0263	
A_87_P115118	0.03057	2.098877	up	458.5415	342.3074	
A_87_P088946	0.00375	2.241603	up	448.6629	312.2088	
A_87_P023735	0.008586	2.370335	up	432.2021	284.0674	CA5B
A_87_P169628	0.047119	2.073145	up	403.7709	305.6135	APC2
A_87_P021960	0.026986	2.007402	up	403.451	316.2714	TMEM16A
A_87_P000312	0.002176	2.137232	up	372.4648	271.8559	COL22A1
A_87_P308042	0.012653	2.188258	up	350.3798	249.8521	CHD7
A_87_P225408	0.007312	2.801035	up	338.4166	187.8141	SLC23A1
A_87_P011329	0.001563	2.005109	up	330.2014	257.0186	MACF1
A_87_P027387	0.031404	2.028113	up	312.8605	239.3172	LOC770787
A_87_P073186	0.034485	2.219931	up	287.0286	202.2863	
A_87_P015822	0.023102	2.614387	up	274.3423	164.0744	GROUP POLY(I:C)AL3
A_87_P015055	0.037505	3.243896	up	265.8974	124.9142	
A_87_P033365	0.01548	2.274323	up	255.4082	175.5869	

A_87_P037881	0.0132	2.54134	up	253.1551	154.6555	SOUL
A_87_P022624	0.018903	2.095799	up	251.8418	188.1497	
A_87_P011977	0.007772	2.044948	up	247.4453	188.718	CCDC132
A_87_P016435	0.009102	2.119412	up	221.1672	163.1128	SACS
A_87_P105108	0.011071	2.182471	up	211.8441	151.1036	TRPS1
A_87_P022669	0.006365	2.004091	up	193.1924	150.6822	
A_87_P072551	0.03794	2.446547	up	190.3246	119.4279	SLC23A1
A_87_P122043	0.015911	3.100986	up	187.8896	95.28166	PTPRF
A_87_P104513	0.019273	2.329722	up	186.4844	124.6192	MYBL1
A_87_P018117	0.045994	2.514816	up	174.9205	106.3156	
A_87_P036003	0.00641	3.325646	up	156.5304	73.68145	CHTF18
A_87_P102461	0.023168	2.373017	up	146.217	95.9227	LOC776557
A_87_P035829	0.00229	2.786086	up	132.1193	73.95721	F2RL1
A_87_P116918	0.010106	2.98085	up	127.1796	66.79111	
A_87_P087346	0.035268	2.007636	up	126.6924	98.15745	LOC425228
A_87_P125993	0.02429	2.768192	up	122.2763	69.94974	GRIA3
A_87_P012660	0.039436	2.004756	up	107.6599	84.48067	
A_87_P028170	0.006212	2.028803	up	103.0666	79.49775	KREMEN1

Table S3-2 Downregulation of differentially expressed genes in the spleen in group Poly(I:C) with FC≥2 and Signal Density of Raw Data ≥100.

ProbeName	p-value	|FC|	Regulation	GROUP POLY(I:C)(RAW_DATA)	CONTROL(RAW_DATA)	GeneSymbol
A_87_P263263	0.028744	2.441037	down	19135.81	72900.14	RSFR
A_87_P036207	0.00438	2.090177	down	3328.199	10873.39	C3AR1
A_87_P065896	0.01387	2.237272	down	2799.326	9824.017	
A_87_P055536	0.010562	2.07731	down	2473.896	8040.143	SLBP
A_87_P140493	0.012572	2.05147	down	2469.863	7889.899	TLR16
A_87_P058561	0.01154	2.000389	down	2462.008	7668.414	TLR6
A_87_P037907	0.005948	2.126369	down	1933.563	6433.736	SDHA
A_87_P193683	0.025462	2.009378	down	1744.57	5435.123	ASL1
A_87_P234808	0.048501	2.210926	down	1732.258	5893.944	CTSS
A_87_P072881	0.008154	3.689032	down	1019.39	5897.244	ARL10
A_87_P035265	0.01682	2.117106	down	723.5488	2404.719	CDKN2A
A_87_P107113	0.007747	2.062304	down	711.5373	2293.686	PLBD1
A_87_P106828	0.048876	2.119471	down	606.9369	1982.214	TMEM140
A_87_P067731	0.041078	2.149046	down	576.5978	1927.181	
A_87_P008724	0.010385	2.029664	down	462.3438	1468.822	K123
A_87_P022808	0.014186	2.046842	down	401.2164	1278.45	MOG
A_87_P008690	0.005182	2.585869	down	398.9553	1613.69	GCH1
A_87_P064081	0.001517	2.151078	down	386.292	1298.169	STX12
A_87_P240163	0.009681	2.053077	down	376.8029	1204.993	MOG
A_87_P156733	0.00434	2.299787	down	350.1905	1258.702	STX12

A_87_P193738	0.041475	2.611675	down	339.6667	1385.975	LOC771069
A_87_P019694	0.008127	3.497982	down	297.4808	1635.84	LYG2
A_87_P297403	0.005567	2.111812	down	225.356	742.1928	NPL
A_87_P008921	0.031571	2.03715	down	214.3319	676.5514	HMOX1
A_87_P065486	0.022738	2.652414	down	212.2025	886.1909	LOC771069
A_87_P023106	0.031828	2.573704	down	201.0348	815.4284	PLBD1
A_87_P021050	0.015256	2.092198	down	169.0554	551.4033	
A_87_P263168	0.048082	2.655546	down	155.2642	649.6974	PLBD1
A_87_P021085	0.017394	2.644538	down	149.0411	615.71	PLCXD1
A_87_P030656	0.004543	2.298788	down	126.5286	454.2228	LOC769044

Table S4-1 Upregulation of differentially expressed genes in the caecum in group SP with FC≥2 and Signal Density of Raw Data ≥100.

ProbeName	p-value	|FC|	Regulation	GROUP SP(RAW_DATA)	CONTROL(RAW_DATA)	GeneSymbol
A_87_P017067	0.014833	2.241353	up	27158.97	10506.86	CLEC3B
A_87_P055986	0.026444	2.048135	up	9172.174	3898.472	PDLIM3
A_87_P110393	0.044918	2.134904	up	8211.222	3363.067	FHL2
A_87_P193418	0.046498	2.219647	up	7528.244	2944.842	FHL2
A_87_P213848	0.026094	2.937232	up	6073.972	1757.178	LOC423423
A_87_P265788	0.047425	2.028906	up	5994.927	2552.497	BAG2
A_87_P055721	0.004181	2.57088	up	5538.785	1861.226	HOXD11
A_87_P009294	0.004336	2.365375	up	3699.251	1356.083	HOXD11
A_87_P080001	0.006183	2.206574	up	2488.212	976.5921	CISD1
A_87_P245708	0.033453	2.915283	up	2122.973	633.1538	ANLN
A_87_P034042	0.040937	4.771468	up	2106.639	398.8331	ACTC1
A_87_P051766	0.046243	2.132916	up	1784.825	733.5394	LOC418424
A_87_P151068	0.008562	2.248188	up	1701.529	656.2959	MAMDC2
A_87_P037492	0.021	2.189082	up	1638.75	647.6197	PDLIM3
A_87_P009048	0.026677	2.046262	up	1590.921	674.5408	CENPF
A_87_P085056	0.046133	2.137321	up	1406.97	564.0237	SCG5
A_87_P092561	0.009984	2.567488	up	1324.834	446.0624	MAMDC2
A_87_P114838	0.016724	3.038796	up	1122.808	320.8924	
A_87_P014112	0.020187	2.337636	up	932.2706	347.1294	
A_87_P152008	0.004968	2.519559	up	775.9677	266.9718	KLHDC8A
A_87_P016444	0.016629	2.245398	up	760.8056	292.9314	
A_87_P027905	0.019624	2.050455	up	704.9199	299.1262	COMP
A_87_P084681	0.037432	2.011036	up	652.0169	279.2924	
A_87_P121843	4.89E-04	2.784071	up	562.507	175.1498	
A_87_P054746	0.021351	2.122426	up	545.0016	223.2091	CENPF
A_87_P102401	0.003087	2.933887	up	352.884	104.1954	ANLN
A_87_P024218	0.017541	2.21491	up	335.0634	132.1076	
A_87_P236533	0.046516	7.077659	up	318.3763	37.00138	C20H20orf85

A_87_P104513	0.002662	3.046689	up	315.2515	89.91791	MYBL1
A_87_P083891	0.045104	2.080555	up	312.0908	129.332	
A_87_P179618	0.018637	3.150793	up	305.2546	84.68122	LOC768920
A_87_P319922	0.024433	3.271542	up	299.3379	78.5472	LOC768920
A_87_P022300	0.046964	2.042768	up	291.6057	122.5599	RIC3
A_87_P145383	0.039066	2.329995	up	282.5269	105.3242	SLIT1
A_87_P032570	0.043419	2.858929	up	247.6722	73.61479	
A_87_P122863	0.032068	6.574216	up	246.7759	33.84986	
A_87_P104908	0.009377	2.041087	up	208.8738	88.86916	OSR2
A_87_P133328	0.001833	2.051884	up	201.0862	84.97775	
A_87_P021436	0.04428	2.539008	up	183.9284	63.00039	TUB
A_87_P037697	0.043356	3.221175	up	163.8235	45.27623	COL20A1
A_87_P014431	0.030014	2.533612	up	162.5813	56.09332	
A_87_P011901	0.002521	2.03188	up	161.1629	68.68959	
A_87_P296773	0.039883	2.340923	up	156.9485	58.23907	ALDH1L2
A_87_P037881	0.00596	3.139463	up	155.8588	43.17721	SOUL
A_87_P018115	0.041883	2.215137	up	152.6586	58.6579	
A_87_P053201	0.026585	2.044309	up	141.0832	59.69648	SYNPR
A_87_P037934	0.034472	8.460807	up	139.0743	15.11028	GDF5
A_87_P056771	0.021345	2.403871	up	131.1553	47.09236	GLP1R
A_87_P012994	0.011458	2.221206	up	123.0653	47.83742	
A_87_P106548	0.033798	2.003954	up	122.4247	53.13174	TRHDE
A_87_P101681	0.006572	2.674664	up	117.2865	37.99509	DGKB
A_87_P054406	0.029114	2.063656	up	110.2237	46.68424	OXTR

Table S4-2 Downregulation of differentially expressed genes in the caecum in group SP with FC≥2 and Signal Density of Raw Data ≥100.

ProbeName	p-value	|FC|	Regulation	GROUP SP(RAW_DATA)	CONTROL(RAW_DATA)	GeneSymbol
A_87_P081341	0.008753	2.100018	down	5147.149	9383.597	LOC423942
A_87_P124573	0.018046	2.063123	down	4147.323	7464.053	MOV10
A_87_P034814	0.007111	2.76483	down	2803.302	6681.683	IFIT5
A_87_P081336	0.008268	2.142918	down	2141.293	3960.148	LOC423942
A_87_P275528	0.028744	2.018683	down	1590.584	2771.403	PSAP
A_87_P094106	0.046616	2.009193	down	998.6245	1720.768	GPR112
A_87_P315787	0.01107	2.017383	down	404.4446	704.7305	PARP14
A_87_P150528	0.031356	2.081949	down	399.3919	724.4912	
A_87_P220458	0.026499	2.00207	down	397.9784	687.4885	
A_87_P260193	0.034129	2.359568	down	337.3372	698.2982	
A_87_P090556	0.015171	2.133231	down	227.6391	420.3683	
A_87_P129690	0.012764	2.245596	down	218.1902	424.9018	CATHL3
A_87_P017222	0.022391	2.159531	down	196.3387	370.0386	
A_87_P073026	0.004324	2.827911	down	173.2249	423.5829	

A_87_P263168	0.027271	2.157093	down	171.706	317.8527	PLBD1
A_87_P018024	0.028947	12.86841	down	147.5546	1823.653	CD72
A_87_P022808	0.042314	2.469146	down	122.4936	258.4686	MOG
A_87_P016668	0.039132	2.424182	down	118.4504	252.0765	CILP

Table S5-1 Upregulation of differentially expressed genes in the caecum in group SE with FC≥2 and Signal Density of Raw Data ≥100.

ProbeName	p-value	|FC|	Regulation	GROUP SE(RAW_DATA)	CONTROL(RAW_DATA)	GeneSymbol
A_87_P014973	0.020842	17.84019	up	1016.454	49.31717	
A_87_P167163	0.004804	2.085917	up	693.1338	300.2516	
A_87_P324787	0.028186	2.833615	up	84509.34	26702.72	LOC771291
A_87_P105598	0.014049	2.085026	up	129.2328	55.79695	
A_87_P262868	0.040523	2.148635	up	939.7851	391.43	PDE9A
A_87_P093371	0.008223	2.488489	up	327.6603	118.3814	LINGO2
A_87_P050041	0.036331	3.017808	up	102.1938	31.13181	SLC18A3
A_87_P078321	0.026316	2.008928	up	3539.299	1589.067	RGS5
A_87_P012632	0.017713	2.076617	up	4341.877	1871.128	
A_87_P055721	0.002503	2.051615	up	4243.532	1861.226	HOXD11
A_87_P023396	0.045685	2.151921	up	5636.413	2364.565	SLC5A9
A_87_P146423	0.039847	2.864879	up	134180.9	41637.73	MT4
A_87_P015825	0.036988	2.103959	up	1296.175	559.201	PPP1R14C
A_87_P007845	0.009348	2.734872	up	663.7703	217.8311	LOC771291
A_87_P037881	0.031403	2.989617	up	143.7049	43.17721	SOUL
A_87_P013037	0.046313	2.03394	up	4559.297	2033.95	ABHD6
A_87_P055036	0.027605	5.717663	up	409.6541	66.92852	CL2
A_87_P028710	0.006318	4.145064	up	10236.29	2232.66	SLC13A2
A_87_P202443	0.04063	3.39792	up	105451.5	27249.55	MT4
A_87_P024407	0.012672	16.21543	up	162.0195	8.613574	SEBOX
A_87_P023409	0.048755	5.266966	up	299.0122	48.23381	NSF
A_87_P096116	0.004699	3.170034	up	2761.577	786.9977	RASL11B

Table S5-2 Downregulation of differentially expressed genes in the caecum in group SE with FC≥2 and Signal Density of Raw Data ≥100.

ProbeName	p-value	|FC|	Regulation	GROUP SE(RAW_DATA)	CONTROL(RAW_DATA)	GeneSymbol
A_87_P091316	0.026362	2.798214	down	132.564	339.9412	LOC770431
A_87_P129978	0.034031	2.929382	down	332.9392	889.0701	LOC426330
A_87_P090256	0.04415	2.375634	down	136.039	295.3577	CHIR-AB1
A_87_P037011	0.017321	2.244148	down	272.4023	553.4141	NCF1
A_87_P117488	0.04391	2.026281	down	957.6114	1751.608	SLC31A2

A_87_P129888	0.048704	2.413237	down	625.6784	1350.403	
A_87_P116628	0.03748	2.422412	down	3052.228	6712.746	CLIC2
A_87_P038224	0.023787	2.275201	down	142.9307	292.5867	PSAP
A_87_P063616	0.031775	2.251122	down	3384.81	6916.481	LOC771877
A_87_P129963	0.02664	2.896826	down	462.0664	1209.644	
A_87_P195668	0.041027	2.269247	down	9640.238	19603.02	CTSH
A_87_P090306	0.025731	2.428305	down	118.2441	262.0523	LOC425113
A_87_P022311	0.031961	2.097193	down	641.5574	1211.964	CNRIP1
A_87_P082566	0.039744	2.490633	down	147.1333	327.6644	FAP
A_87_P134688	0.036334	2.406621	down	1609.516	3554.54	C20orf42
A_87_P170028	0.02129	2.418963	down	142.0877	312.0223	
A_87_P028667	0.040998	3.16637	down	302.4254	884.3738	CENTA2
A_87_P091001	0.042836	2.133442	down	230.8228	442.3807	LOC771258
A_87_P021759	0.036146	3.010039	down	311.5055	843.6882	LOC417192
A_87_P132743	0.002793	2.438193	down	310.1846	682.4832	
A_87_P132785	0.00624	3.196407	down	469.2683	1352.766	
A_87_P250273	0.034193	2.13936	down	294.4136	573.1051	ITGB2
A_87_P149603	0.049236	2.823932	down	501.6462	1265.674	LOC421099
A_87_P122853	0.006265	2.158789	down	4384.928	8504.026	
A_87_P132808	0.017809	2.929734	down	573.1385	1509.634	
A_87_P014773	0.031846	3.321809	down	169.1831	518.9665	
A_87_P065896	0.040972	3.256397	down	1213.196	3590.312	
A_87_P035946	0.026552	2.846739	down	806.4634	2101.139	GSDMA
A_87_P147948	0.040886	3.264982	down	711.2198	2118.651	LOC771877
A_87_P008850	0.031357	2.22686	down	248.4611	504.1104	ITGB2
A_87_P092256	0.037905	2.159178	down	119.2861	234.1683	LOC769117
A_87_P009677	0.047268	2.704191	down	4235.871	10169.62	DNASE2B
A_87_P132768	0.048015	3.032471	down	523.8539	1420.796	
A_87_P058371	0.035593	2.56755	down	1164.157	2708.206	TLR4
A_87_P022555	0.018513	2.496512	down	5844.545	13280.83	C1QA
A_87_P012535	0.033289	2.120312	down	151.6456	285.7086	FCN2
A_87_P086412	0.024455	2.712421	down	442.2019	1095.675	
A_87_P036207	0.037717	3.275309	down	2033.046	6092.049	C3AR1
A_87_P025636	0.00897	2.237158	down	191.6863	388.3333	
A_87_P089381	0.033139	2.799693	down	817.3422	2097.225	
A_87_P310842	0.010904	2.996206	down	297.6038	802.3868	
A_87_P051526	0.049502	3.292744	down	10625.37	32478.58	LOC417536
A_87_P263263	0.016489	5.333937	down	9668.349	45843.88	RSFR
A_87_P087212	0.043692	2.304121	down	782.1324	1638.964	
A_87_P220458	0.02558	2.23014	down	341.6007	687.4885	
A_87_P008872	0.006767	4.21702	down	38542.8	146529.7	RSFR
A_87_P058641	0.040571	3.017167	down	550.9206	1500.559	CHIR-B3
A_87_P087131	0.012217	2.650401	down	517.3791	1235.945	
A_87_P221583	0.00117	2.097165	down	131.7905	249.306	FAP

A_87_P132858	0.047916	3.172536	down	377.8083	1074.51	
A_87_P058646	0.032244	2.069559	down	366.9934	683.4158	CHIR-B4
A_87_P056446	0.033853	2.234042	down	1280.009	2581.117	ACSL1
A_87_P076361	0.041976	2.141753	down	122.0077	238.6055	LOC770352
A_87_P130018	0.046061	2.697001	down	307.9935	748.9995	
A_87_P078111	0.006525	2.496402	down	569.6565	1283.287	P2RY13
A_87_P132888	0.049218	3.858826	down	834.7884	2805.653	FCER1G
A_87_P050996	0.015158	10.26013	down	856.0988	7853.291	B-G
A_87_P129778	0.028192	3.036991	down	462.6163	1269.174	
A_87_P306688	0.026473	2.971798	down	398.3714	1062.017	CHIR-B3
A_87_P014908	0.031702	3.435949	down	136.5117	434.6087	
A_87_P091306	0.030058	2.644811	down	154.2082	370.8103	
A_87_P020905	0.033782	3.723495	down	5275.214	17357.5	FCER1G
A_87_P091081	0.017436	3.062251	down	659.7219	1823.375	LOC426330

Table S6-1 Upregulation of differentially expressed genes in the caecum in group Poly(I:C) with FC≥2 and Signal Density of Raw Data ≥100.

ProbeName	p-value	|FC|	Regulation	GROUP POLY(I:C)(RAW_DATA)	CONTROL(RAW_DATA)	GeneSymbol
A_87_P037391	0.006664	2.703767	up	29518.29	10619.04	SULT1C3
A_87_P213848	1.68E-04	2.113179	up	3804.491	1757.178	LOC423423
A_87_P051766	0.035518	2.326937	up	1730.728	733.5394	LOC418424
A_87_P063816	0.004375	2.108062	up	1169.931	540.9439	LOC419892
A_87_P050036	0.013709	2.058164	up	820.5958	388.3044	SOCS2
A_87_P232208	0.008595	2.03741	up	456.0847	218.6075	SOCS2
A_87_P102401	0.011823	2.133296	up	228.08	104.1954	ANLN
A_87_P104513	0.007677	2.382035	up	219.1868	89.91791	MYBL1
A_87_P179618	0.029496	2.106558	up	180.8519	84.68122	LOC768920
A_87_P236533	0.034036	3.508875	up	132.3831	37.00138	C20H20orf85

Table S6-2 Downregulation of differentially expressed genes in the caecum in group Poly(I:C) with FC≥2 and Signal Density of Raw Data ≥100.

ProbeName	p-value	|FC|	Regulation	GROUP POLY(I:C)(RAW_DATA)	CONTROL(RAW_DATA)	GeneSymbol
A_87_P035933	0.039469	2.215196	down	8762.724	19238.58	NFKBIZ
A_87_P111728	0.02805	2.012913	down	3700.156	7311.152	CTSC
A_87_P124573	0.021516	2.234402	down	3407.894	7464.053	MOV10
A_87_P017535	0.044468	2.302525	down	3098.57	6972.997	
A_87_P024462	0.042179	2.211759	down	2577.747	5534.331	
A_87_P174103	0.012048	2.205112	down	2432.484	5247.165	P2RX7
A_87_P034814	0.001515	3.21136	down	2133.253	6681.683	IFIT5
A_87_P021556	0.019185	2.014075	down	1571.214	3097.13	LOC416147
A_87_P217653	0.026853	2.26771	down	1182.391	2619.817	STAT1

A_87_P072881	0.018247	3.137558	down	737.279	2243.917	ARL10
A_87_P110888	0.049583	2.248944	down	623.6538	1351.796	EPSTI1
A_87_P106828	0.036053	2.202654	down	620.0512	1317.762	TMEM140
A_87_P211953	0.019617	2.090299	down	510.0576	1039.841	STAT1
A_87_P081091	0.035285	2.37391	down	501.936	1147.516	COL17A1
A_87_P101556	0.030013	2.97034	down	460.8398	1318.066	SAMD9L
A_87_P115188	0.00655	2.287285	down	433.1795	966.6478	STAT1
A_87_P315787	0.0021	2.042609	down	353.3085	704.7305	PARP14
A_87_P019522	0.025044	2.451614	down	315.7911	760.3036	
A_87_P105043	0.017475	4.458349	down	309.3151	1324.275	
A_87_P014410	0.025478	2.959571	down	290.5617	840.4594	
A_87_P009753	0.022865	10.47075	down	177.3527	1703.509	LOC420300
A_87_P299338	0.00329	2.720574	down	153.426	407.0948	SAMD9L
A_87_P133573	0.011658	2.170783	down	123.9796	264.01	CRTAM
A_87_P015250	0.002682	3.436842	down	121.2566	406.9168	
A_87_P030818	0.021311	2.825544	down	120.5811	335.4609	
A_87_P161603	0.009158	2.850308	down	111.6946	309.99	
A_87_P110193	0.005713	2.19285	down	109.8248	234.6588	PLCXD1

Table S7 qRT-PCR primers for 5 target genes.

Gene names	Direction	Primer	Tm (℃)
RSFR	F	AAGAAGAAGAAGGAAGAAGAG	59.7
	R	GAACACCATAGCACATAGAC	60.8
VPREB3	F	GAAGACAACGGTCGCTATT	62.9
	R	TTGAGGGCAAATCTTTATTACATT	61.6
CD72	F	AATATGAGCAGCGTATCAGA	61.5
	R	TATTGCCAAGTATCAAGGATG	60.5
IL15	F	GATTCTGTTCTTCTGTTCTGAG	61.3
	R	TTGGTACATAAGCACATAGGA	61.3
GAL1	F	GTTCTTACTTCCTTGCTGTA	59.9
	R	CATTTCCCACTGATGAGAGT	62.2

Table S8 Statistical description of the difference in the expression of genes in each group in the spleen

Group	No. of DEGs	Upregulated probes	Proportion of up-regulated probes	Down-regulated probes	Proportion of down-regulated probes
SE	585	383	65.47%	202	34.53%
SP	743	508	68.37%	235	31.63%
Poly(I:C)	903	579	64.12%	324	35.88%

Table S9 Statistical description of the difference in the expression of genes in each group in the caecum.

Group	No. of DEGs	upregulated probes	Proportion of upregulated probes	Downregulated Probes	Proportion of downregulated probes
SE	384	162	42.19%	222	57.81%
SP	488	285	58.40%	203	41.60%
Poly(I:C)	296	129	43.58%	167	56.42%
